# Why are some countries rich and others poor? development and validation of the attributions for Cross-Country Inequality Scale (ACIS)

**DOI:** 10.1371/journal.pone.0298222

**Published:** 2024-02-27

**Authors:** Michela Vezzoli, Roberta Rosa Valtorta, Attila Gáspár, Carmen Cervone, Federica Durante, Anne Maass, Caterina Suitner

**Affiliations:** 1 Department of Psychology, University of Milano-Bicocca, Milan, Italy; 2 Department of Developmental Psychology and Socialisation, University of Padua, Padua, Italy; 3 Institute of Economics (KRTK-KTI), Centre for Economic and Regional Studies, Budapest, Hungary; 4 Psychology Program, Division of Science, NYU Abu Dhabi, Abu Dhabi, United Arab Emirates; 5 University of Padua, Padua, Italy; University of Padova, ITALY

## Abstract

Understanding lay theories on the causes of economic inequality is the first step to comprehending why people tolerate, justify, or react against it. Accordingly, this paper aims to develop and validate with two cross-sectional studies the Attributions for Cross-Country Inequality Scale (ACIS), which assesses how people explain cross-country economic inequality–namely, the uneven distribution of income and wealth between poor and rich countries. After selecting and adapting items from existing scales of attributions for poverty and wealth, in Study 1, we tested the factorial structure of this initial pool of items in three countries with different levels of economic development and inequality, namely, Italy (*n* = 246), the UK (*n* = 248), and South Africa (*n* = 228). Three causal dimensions emerged from the Exploratory Factor Analysis: “rich countries” (blaming the systematic advantage of and exploitation by rich countries), “poor countries” (blaming the dispositional inadequacy and faults of poor countries), and “fate” (blaming destiny and luck). The retained items were administered in Study 2 to three new samples from Italy (*n* = 239), the UK (*n* = 249), and South Africa (*n* = 248). Confirmatory Factor Analysis (CFA) corroborated the factorial structure of the ACIS, and Multi-Group CFA supported configural and metric invariances of the scale across countries. In addition, we show internal consistency and construct validity of the scale: the scale correlates with relevant constructs (e.g., beliefs about cross-country inequality and ideological orientation) and attitudes toward relevant policies related to international redistribution and migration. Overall, the scale is a valid instrument to assess causal attribution for cross-national inequality and is reliable across countries. By focusing on resource distribution from an international perspective, this scale will allow researchers to broaden the discussion on economic inequality to a global level.

## Introduction

The economic gap between rich and poor countries (e.g., average income differences between, say, Italy and India) is considerable despite the fact that emerging countries have experienced an upward surge in recent decades (United Nations, 2021). It was estimated that the average annual income in South and Southeast Asia is 50% of the € 16,700 global average Purchasing Power Parity, while in Sub-Saharan Africa it is 31% [1]. Latin America, Russia, and East and Central Asia have average incomes close to the global average. In Europe, the average income is more than twice the global average, while in North America it is three times the global average. This means that, for example, Europeans earn twice as much as East Asians, while North Americans earn, on average, 6 to 10 times more than Sub-Saharan Africans and South- and South-east Asians. The difference between rich and poor countries is even more impressive if we look at income earned per hour worked, given that Sub-Saharan Africans and South- and South-east Asians spend around 30% more time at work than Europeans and North Americans [1].

In the sociopsychological literature, economic inequality has largely been studied within countries [2–4], mainly neglecting a global perspective. Yet, we live in a highly unequal world and this great difference in wealth and income between countries is self-evident: In rich countries, people are healthier and live longer, they are much better educated and have access to a range of amenities and options in life that are precluded to people in poor countries [5]. According to several international organizations, including the International Organization for Migration of the United Nations [6], these global inequalities are often the trigger for migration waves, as people try to improve their quality of life by moving to more affluent countries that offer better labor opportunities, healthcare, and education [7]. Moreover, global inequality is also associated with international conflicts, climate issues, and the possibility of effectively managing global health threats, such as pandemics [8,9].

As such, tackling global inequality is not only of interest to poor countries, since a reduced gap would also be beneficial to wealthier nations [10]. As a result of globalization, we are all interconnected; problems such as poverty, climate change, and migration are never confined to one country alone, and even the richest countries have people living in poverty. Given the high levels of cross-country inequality and considering that it accounts for 32% of global economic inequality (i.e., inequality among all individuals on earth; [1]), reducing global inequality is a central point on the agenda of many international organizations, such as the United Nations [11], the European Union [12], and the World Economic Forum [13]. To diminish economic disparities between countries, these organizations acknowledge the importance of implementing policies aimed at a more responsible management of migration flows, ensuring special and dedicated treatment for developing nations, and encouraging investments in these countries. For these policies to be supported by the population, it is critical to investigate the lay theories through which people explain the economic gap between countries, which leads to the perception of what kind of social change is needed [14].

Understanding how people explain inequality is relevant because it is closely intertwined with the degree to which people tolerate inequality. For example, endorsing situational causes of poverty and wealth is related to individuals’ aversion to economic inequality [15], perceived lack of economic mobility [16], and the amount of inequality an individual perceives as fair [17] or as immoral and outraging [18,19]. Moreover, different causal explanations of poverty influence how people view the poor [20] and whether they believe economic resources should be shared with those who need them. Individuals who believe that situational factors cause poverty are more likely to donate [21,22] and to accept spending on social security and social protection policies than those who attribute poverty to dispositional causes [23–26]. Experimental studies have replicated this pattern [27,28] and established that interventions on poverty perception (e.g., through an interactive virtual simulation of poverty) promote situational attributions for poverty, which, in turn, increase support for redistribution policies and prosocial behaviors (e.g., donations) [15]. As of now, however, the literature lacks an instrument to assess causal attributions for cross-country inequality. Thus, providing the scientific community with a new tool to measure how people make sense of cross-country economic inequality is useful and timely as it may advance our knowledge of how individuals interpret and react to global phenomena.

This paper aims to develop and validate a new tool, the Attributions for Cross-Country Inequality Scale (ACIS), designed to measure individuals’ perceptions of the causes of economic inequality between nations. We first generated items using a top-down approach. Then, we conducted two correlational studies to test the factorial structure of the scale through exploratory (Study 1) and confirmatory (Study 2) factor analytic approaches. We assessed criterion validity via correlational analyses between the ACIS factors and relevant socio-psychological constructs, such as judgments of and reactions to inequality (i.e., unfairness, outrage, and immorality) and ideological orientations (i.e., social dominance orientation, economic system justification, meritocratic beliefs, and political orientation).

Below, we will review the literature and current measures of causal attributions for poverty and wealth to highlight the ways in which a scale such as ours would contribute to the literature on economic inequality: (a) by validating this new measure across low- and high-income countries; (b) by focusing on economic inequality rather than poverty and wealth; and (c) by providing evidence on the lay theories about cross-country, rather than domestic, inequality.

### Causal attributions for economic inequality

#### Current measures of causal attributions

Attribution theory posits that people attempt to explain events and features of the social world in causal terms and that these causal attributions influence people’s psychosocial processes and behaviors [29–31]. For instance, perceiving someone to be at least partially responsible for their misfortune reduces sympathy, willingness to help, and help-giving [30,32].

Much of the existing research on the causal attributions for inequality investigated laypeople’s perceived causes of domestic poverty and, to a lesser extent, domestic wealth [33–36]. Usually, these studies identify three forms of attributions: *dispositional causes* (i.e., causes attributed to the poor themselves, such as lack of effort or loose morals), *situational causes* (i.e., social and economic factors), and *fatalistic causes* (e.g., luck and fate). This tripartite model has received empirical support from various studies [37–40], across several Western, Educated, Industrialized, Rich and Democratic societies (WEIRD) [41,42] and non-WEIRD countries [43,44]. Further support to this model comes from studies that investigated the causal attributions for poverty in developing countries [45,46].

Importantly, situational and dispositional attributions are not opposing dimensions: both can be endorsed simultaneously [17], and attributions on one dimension can be shifted without corresponding influence on the other [15]. While these three sets of attributions emerge consistently across cultures, their relative weight varies. Indeed, dispositional causes of poverty are the most endorsed in Western cultures [47–49], while situational and, to a lesser extent, fatalistic causes are prevalent in non-Western cultures [37,38,50]. However, it should be noted that while many studies using Feagin’s items produced a relatively stable pattern of results, research with different but similar sets of items shows greater complexity [51]. While dispositional and societal dimensions emerge consistently across measures, other research has identified another factor, namely cultural attributions [20,52,53], which are interpreted as “culture of poverty” beliefs (e.g., breakdown of the nuclear family, being born into poverty). Therefore, there is still a lack in the literature of a validated scale that can assess attributions for economic conditions equally across low-income and high-income countries, and even more so one that focuses specifically on inequality, as detailed below.

#### The importance of inequality

How people perceive and judge national economic inequality and what people think about the causes of inequality, have been central questions in economics for decades (see [54] for a compelling popular narrative). Economists usually look at the problem from a policy perspective: People in some countries care much more about economic inequality than in others, even when the countries’ levels of economic development are otherwise comparable. One exemplary difference emerges between North America and Western Europe societies [55]: Americans live in a much more unequal society than most Europeans and they also care much less about economic inequality.

This might happen because a rational person’s judgment on economic inequality, argue economists, depends on how they evaluate the status quo; how they expect it to affect them; how different it is from what they would perceive as ideal; and what they expect they can do about it. Even when the current distribution is unfavorable for someone, they might accept inequality if they perceive that they (or their children) will be able to climb the ladder and become beneficiaries of the status quo (see e.g., [56–58]). As they base their beliefs in social mobility on their own experiences, heterogeneous beliefs may persist in a society [59]. In line with this reasoning, perceived social mobility affects preference for redistribution [60]. Interestingly, the more unequal a society, the more its members tend to believe, on average, in meritocratic values and the role of effort [61].

Alesina and colleagues [62] showed evidence form a cross-country survey that people in different countries systematically differ in how much importance they attribute to luck in someone’s income, and by extension, in income inequality. They found that while only 30% of Americans believe that income is determined by luck, twice as many Europeans do so, and this strongly correlates with the rate of redistribution across these countries. Theoretical economic arguments have suggested that, rather than reflecting inherent differences across citizens of different nations, these differences reflect diverging collective beliefs that reinforce themselves (i.e., [63,64]). On the one hand, recent large-scale experimental evidence supports systematic variations in collective beliefs about the source of income inequality (i.e., individual productivity and effort vs luck) and in fairness views between the US and Scandinavia (see [65]). Along the same lines, experimental evidence shows that when individuals are informed about the actual determinants of income, Europeans and Americans are not different in how much they are willing to redistribute [66]. Cross-cultural similarities are also observed in the cost attributed to inequality reduction [65].

Collective beliefs are not necessarily correct: Alesina and colleagues [60] show that while Americans are rightfully more optimistic about social mobility than Europeans, as social mobility is faster in the US, the former are actually too optimistic (overestimating mobility), while the latter are too pessimistic (underestimating mobility). The beliefs themselves are, however, passed on across generations: Gartner, Mollerstrom, and Seim [67] show that parents emphasize the role of effort to their children to a greater extent than they actually believe.

Whether or not correcting these beliefs changes support for redistribution also depends on the ideological leaning of the individual (i.e., liberals think the government can reduce inequality, conservatives believe that the government is part of the problem, see [60]. Interestingly, differences in the willingness to redistribute wealth or income varies across countries (in this case, US vs Norway) and these differences seem to be driven by fairness, rather than by efficiency or cost, considerations [65].

Beliefs can be self-serving and motivated. On the one hand, factors such as race are likely to inform redistributional preferences. For instance, people are more reluctant to decrease inequality if the poor are of a different (rather than same) ethnicity [62]. On the other hand, people deem inequality in their ingroups much more unfair, while at the same time underestimating the actual inequalities in these groups [68].

Experimental evidence has also demonstrated that people tend to endorse beliefs that benefit themselves. Deffains and colleagues [69] show that participants are more likely to attribute success to effort (rather than luck) when they themselves are successful, and less likely to desire redistribution. Moreover, people choose to remain ignorant about the actual causes of inequality when confronted with information that would challenge their (meritocratic) beliefs about their own personal success [70].

Social psychologists have also been investigating the perception of inequality, from a rather different angle than individual rationality. Kraus and colleagues [71] rated the importance of *situational* (e.g., structures of inheritance or discrimination) and *dispositional* (e.g., hard work and effort) factors in explaining growing (vs. decreasing) economic disparity in the US. However, as the scale factorial structure was not tested, it remains unclear whether the tripartite model can also be applied to domestic inequality and, crucially, cross-country inequality. Indeed, while poverty and wealth are related to inequality, they are also distinct phenomena [72]. When asked to give opinions about poverty or wealth, individuals may refer to people living in suboptimal or ideal material conditions. When asked to think about inequality, they may consider how society is economically structured in a broader sense. This is crucial to note because inequality is fundamentally an intergroup issue.

Intergroup attribution refers to how members of different social groups explain the behavior and economic conditions (e.g., the socioeconomic standing) of members of their own and other social groups [73]. When differences between groups are to be explained, they might favor the ingroup by blaming the outgroup, or vice-versa. However, there are a few studies that investigated the causal attributions for economic disparities between groups, especially from a cross-country perspective.

### Psychological investigation of cross-country inequality

Economic inequality has attracted the attention of many scholars, including psychologists. While great efforts have been spent on investigating perceptions [74–78] and effects [79–82] of perceived domestic inequality (i.e., inequality within-country), little research has focused on how people perceive cross-country inequality and how it affects psychological processes and behaviors. The few existing studies focused on Western societies (i.e., USA, Germany, France, and the UK) and found that people tend to underestimate how wealthy they are compared to people globally [83–85]. These misperceptions exceed those previously measured in a domestic context, meaning that people are worse at estimating their relative position in the global than in the domestic distribution [83,84]. According to Ziano and Onyeador [85], Westerners’ underestimation of global inequality is driven by a convergence illusion, which is the belief that poorer countries have closed the economic gap with more affluent countries to a larger extent than they actually have.

These studies have also investigated the effects of perceived global inequality on preferences toward global redistribution; the results, however, are mixed. Ziano and Onyeador [85] found that perception of inequality at the global level correlates with support for aid to poorer countries; Fehr and colleagues [83] found that while the usual correlates of national redistributional preferences do predict global preferences among German survey respondents (e.g., luck vs effort beliefs, political orientation), information treatments do not shift the preferences for redistribution (i.e. respondents do not want to give more to the global poor when they learn that themselves are relatively richer in comparison to those poor; [84]).

To further understand the complex relationships between perceptions of cross-country inequality and people’s tolerance of and reactions to it, it is necessary to explore people’s lay theories about why there is an economic gap between countries. Unlike “expert” approaches to explaining inequalities, “lay” attributions are the explanations ordinary people provide to account for inequalities existence and persistence [47]. These explanations can be richer than the two (luck vs effort) usually studied in the economic literature. Indeed, a person who thinks poor countries are poor because they have fewer resources and a person who thinks poor countries are poor because of the history of colonialism would both be categorized as leaning toward “luck” rather than effort, but for rather different reasons, and might support rather different policies. To the best of our knowledge, this is the first work that explores people’s explanations for why there is an economic gap between poor and rich countries across the globe. It does so by providing scholars with a new scale, the ACIS, that measures the level of endorsement of such explanations.

### The current research

We validated the ACIS through two correlational studies aimed at exploring (Study 1) and confirming (Study 2) the factorial structure of the scale and to evaluate its correlations with theoretically relevant psychological constructs.

To further confirm the stability of our scale, we validated the English scale in three different countries, namely Italy (where funding was obtained), the UK (in order to have an English-speaking country with a Gini similar to the Italian one), and South Africa (the country with the highest Gini in the world). Among the three, the UK ranks the highest in terms of GDP per capita (15% above Italy and more than 200% above South Africa, adjusted for purchasing power), while South Africa is by far the most unequal country with a Gini coefficient of .63 compared to the respective values of .352 for Italy and .326 for the UK [86].

In Study 1, Prolific participants were asked to evaluate a list of explanations of cross-country inequality. Factor analysis was performed to define the factorial structure of the scale. Reliability analysis examined its consistency. In Study 2, similar but independent Prolific samples evaluated the selected items. Confirmatory analysis was carried out to test the stability of factorial structure and its cross-cultural invariance. The criterion validity of the ACIS factors was evaluated through correlational patterns with theoretically relevant psychological constructs. In particular, we focused on four main correlates of inequality appraisal, namely political conservatism, social dominance orientation, meritocracy beliefs, and system justification [87]. Specifically, conservatives are generally more likely to explain wealth and poverty in dispositional terms [14,26,32,36,88]. People who prefer hierarchical social structures (i.e., high social dominance orientation; [89] and justify the economic system (i.e., high economic system justification; [90]) are more likely to make dispositional attributions for poverty and wealth than people who score lower on these constructs [91,92]. Finally, people who hold strong meritocracy beliefs tend to attribute the causes of poverty more to dispositional than situational factors [93].

Databases, analysis scripts, and research material can be found online on OSF: https://osf.io/wbdh5/. The reported analyses were executed in R [94]. For the main analyses, we used *psych* [95]; for the Exploratory Factor Analysis (EFA), *lavaan* [96]; for the Confirmatory Factor Analysis (CFA), and *epmr* [97] for reliability analyses.

## Study 1

### Sample and procedure

This study was approved by the Ethical Committee of the University of Padova (protocol number 4558). All participants provided informed written consent prior to study enrollment. We did not have access to information that could identify individual participants during or after data collection. We developed Study 1 on Qualtrics (https://www.qualtrics.com) and distributed it to English-speaking Prolific Academic panelists from Italy, South Africa, and the UK. Prolific workers were paid £2.50 for participating in the study. Data collection took place from February 8th to February 14th, 2022. All participants were informed about the aims of the study and gave their consent before starting the survey. The questionnaire was written in English, and it took approximately 23 minutes to complete. Anonymity was guaranteed as no data allowing the identification of participants was collected. An initial sample of 828 participants was recruited. Specifically, 264 responses were collected from Italy, 290 from South Africa, and 274 from the UK.

After removing participants who did not meet inclusion criteria (i.e., access with a smartphone, n = 35; not fluent in English, *n* = 4; not a citizen or resident of the target country, *n* = 4; failed one or more of the seven attention checks, n = 41; did not finish the questionnaire, *n* = 22), the final sample included 248 participants from the UK, 246 from Italy, and 228 from South Africa. To further ensure the quality of the responses, we checked the geographic location from where the questionnaire was completed and we did not find inconsistencies. Sample sizes were adequate to explore the factorial structure of the ACIS. As Kyriazos [98] indicated, a sample size of at least 200 offers adequate statistical power for measures of up to 40 items [99]. A sensitivity analysis run on G*Power 2 [100] showed that our smallest sample (N = 228) could detect two-tailed, bivariate correlations of r = |.13| with .80 power and α = .05. The descriptive statistics of the three samples are reported in the (S1 Table).

### Measures

The questionnaire had four sections. In the first section, participants provided informed consent to participate in the study. Then, they answered items investigating perceptions, causal explanations, and judgments of domestic and cross-country economic inequality. These were presented in two separate sections counterbalanced across participants (no substantial order effects were observed), and the items were randomized within each scale. This paper presents only data for cross-country inequality, as domestic inequality is irrelevant to the question under investigation. In addition, before presenting the causal explanation items (in both domestic and cross-country economic inequality sections), all participants answered two measures of perceptions of inequality. One was an adaptation to international economic inequality of the widely used "shape of society" question (see International Social Survey Programme; [55]). The other asked participants to estimate the monthly income of people living in richer and poorer countries, relative to those living in middle-income countries. Results, however, showed that these items failed to correlate with the other measures of inequality appraisal included in our study. Since these single items were developed ad hoc and not validated, we took this as an indication of their poor validity, and thus we deemed them unfit to be employed to determine the construct validity of the ACIS; as such, the results are not presented in this paper.

Finally, participants answered ideological measures (e.g., social dominance) and provided socioeconomic (e.g., SSES) and sociodemographic (e.g., gender, age, education) information. The descriptive statistics of the measured variables are reported in S1 Table. All measures were assessed on a 5-point scale (1 = *totally disagree*/*not at all*, 5 = *totally agree*/*very much*), unless otherwise specified.

#### Causal attribution for cross-country economic inequality

To generate the items, we applied a top-down approach based on the tripartite model of causal attributions for domestic poverty, wealth, and Third-World poverty [26,34,36,41,101–105]. The ACIS items were selected and then adapted to the cross-country perspective, which supports the content validity of the scale. The first list of statements was derived by three authors and subsequently discussed with all co-authors. Changes to this list were based on mutual consent, resulting in a final list of 38 statements (see OSF for the full list). Of these, 18 items referenced external and economic factors that exacerbate cross-country inequality (e.g., “Political systems that give special treatment to rich countries,” “The outsourcing of high-tech and manufacturing jobs to developing countries”), also from a historical perspective (e.g., “Decades or even centuries of exploitation by rich countries”); many of these items are original and derived from the economic literature on cross-country inequality [106,107]. Sixteen items referred to the characteristics of poor and rich countries (e.g., “The lack of thrift and proper money management of poor countries,” “The determination of rich countries”). Finally, four items referred to fatalistic causes, such as luck and divine will (e.g., “The good or bad luck the country has compared to other countries”).

#### Attitudes toward cross-country income inequality

Respondents were asked to rate three single-item measures that were retrieved from the International Social Survey Program [108] to tap into their perception of the magnitude of cross-country inequality (*inequality level*; i.e., “Present economic differences between rich and poor countries are too large”), their attitude toward job migration (*migration*; i.e., “People from poor countries should be allowed to work in wealthy countries”), and international redistribution (*redistribution*; i.e., “People in rich countries should make an additional tax contribution to help people in poor countries”). In addition, respondents answered three single-item measures which aimed to tap into perceived unfairness (*unfairness*; i.e., “How fair do you think the income distribution between countries is?”; score reversed), anger (*outrage*; i.e., “How do you feel when you think about economic inequality between nations in the world?”), and *morality appraisal* (i.e., “Would you say that, in general, economic inequality between nations in the world is morally right or wrong?”) of cross-country inequality. Items were adapted from the ISSP V Survey [108].

#### Ideological measures

To assess the beliefs about how much hard work and ability are rewarded and how much people are perceived to deserve their success (i.e., meritocracy), we used the System-Legitimizing Ideology Scale [109] (six items; e.g., “Getting ahead is a matter of working hard and relying on yourself”; α_ITA_ = .84; α_UK_ = .88; α_RSA_ = .79).

To measure the degree to which individuals support group hierarchies, we used the Short Social Dominance Orientation Scale (SDO) [110], which comprises four items (e.g., “We must not push for equality for all groups”; α_ITA_ = .69; α_UK_ = .71; α_RSA_ = .48). Given the low reliability in South Africa, we need to be cautious in interpreting this scale.

To assess the belief that the economic system provides individuals with equal opportunity to succeed and that outcomes are based upon personal deservingness and merit, we used the 12-item version of the Economic System Justification Scale [111] (e.g., “Differences between social classes reflect differences in the natural order of things”; α_ITA_ = .80; α_UK_ = .84; α_RSA_ = .67).

For all these measures we computed the average of the answers, and higher scores indicated stronger beliefs that the world is meritocratic, greater system justification, and greater social dominance orientation.

#### Additional measures

As additional information about participants, we assessed their sense of national identity (average of three items retrieved from ISSP V [108], e.g., “I would rather be a citizen of [country] than of any other country in the world”; α_ITA_ = .69; α_UK_ = .76; α_RSA_ = .66), political orientation (single-item, 1 = *progressive*, 11 = *conservative*), SSES (MacArthur ladder; [112]), and the perceived economic position of their own country (single-item, 1 = *richest 20%*, 2 = *second richest 20%*, 3 = *middle 20%*, 4 = *second poorest 20%*, 5 = *the poorest 20%*). Further, we asked participants how satisfied they were with their life (single-item, 1 = *completely dissatisfied* to 7 = *completely satisfied*) and how much they trusted other people (single-item, 1 = *I need to be very careful in dealing with people*, 4 = *people can almost always be trusted*). All these items were retrieved from the ISSP questionnaire [108]. Finally, participants disclosed their gender (response options: *female*, *male*, and *non-binary*), age, level of education (number of years of formal education) and working status.

### Results

#### Exploratory factor analysis and reliability

To examine the factorial structure of the 38 causal attribution items in the three countries, we conducted EFAs (Maximum Likelihood) with oblique rotation (i.e., oblimin) given that the factors of the scales were not assumed to be orthogonal. The Kaiser-Meyer-Olkin values suggested that the correlation matrices were well suited for EFA (KMO_ITA_ = .87, KMO_RSA_ = .89; KMO_UK_ = .84; [113]). Also, Bartlett’s tests of sphericity were significant at *p* < .001 in all countries (Italy, χ^2^ (703) = 3819.063, *p* < .001; South Africa, χ^2^ (703) = 3072.105, *p* < .001; UK, χ^2^ (703) = 4305.213, *p* < .001). For each EFA, we selected items using the following iterative procedure: First, we ensured that each factor was interpretable and had at least three items [114]. Second, each item had to have standardized factor loadings larger than .40 and communalities larger than .30 [115]. If these conditions were not met, we excluded those items and repeated the procedure, until all the above conditions were met. The final EFA was performed on 19 items and indicated a three-factor model in all three countries. Table 1 presents descriptive information on the items as well as their factor loadings and communalities. The first factor (labeled as *rich countries*) includes those items that attribute the existence of economic inequality between countries to the systematic advantage of and exploitation by rich countries. The second factor (labeled as *poor countries*) comprises those items that attribute the existence of economic inequality to the wrongdoing of developing countries. Finally, the third factor (labeled as *fate*) includes items that attribute economic inequality to fate or divine intervention. Across the three countries, these factors explain more than 50% of the variance. Factors showed satisfactory internal consistency in all countries (α’s between .69 and .91, see Table 1; [116]). Also, the corrected item-total correlations was larger than .30 in all countries (Tabachnick et al., 2019), indicating coherence between any item and the other items composing the same factor.

**Table 1:** EFA of the 19 ACIS items on three factors: Descriptive statistics, reliability, and factor loadings (Study 1).

		Italy						South Africa						UK					
Item		M (SD)	CICT	Rich countries	Poor countries	Fate	*Com*	M (SD)	CICT	Rich countries	Poor countries	Fate	Com	M (SD)	CICT	Rich countries	Poor countries	Fate	*Com*
**1**	Economic systems that create advantages for rich countries	4.23 (1.33)	.67	0.72	-0.10	-0.08	.59	4.54 (0.75)	.57	0.68	-0.03	0.09	.46	4.27 (0.84)	.69	0.72	-0.15	-0.02	.60
**2**	Political systems that give special treatment to rich countries	4.38 (1.39)	.68	0.75	-0.09	0.08	.58	4.48 (0.80)	.57	0.68	0.08	0.00	.46	3.94 (1.06)	.63	0.73	0.09	0.07	.52
**3**	Rich countries deciding the rules of the game	4.34 (1.31)	.66	0.73	0.09	-0.21	.59	4.56 (0.78)	.64	0.74	0.00	-0.03	.55	4.21 (0.98)	.76	0.76	-0.18	0.05	.69
**4**	Decades or even centuries of exploitation by rich countries	4.46 (1.21)	.63	0.68	-0.21	0.05	.54	4.59 (0.83)	.66	0.74	0.00	-0.08	.57	4.27 (0.98)	.73	0.77	-0.09	-0.13	.66
**5**	Rich countries snatch the resources of poor countries	4.30 (1.29)	.59	0.68	-0.04	0.05	.46	4.55 (0.81)	.56	0.64	0.02	-0.16	.46	3.93 (1.09)	.68	0.79	0.11	-0.07	.58
**6**	Greediness of rich countries	4.01 (1.39)	.59	0.72	0.13	-0.01	.49	4.48 (0.82)	.62	0.71	0.02	-0.11	.52	4.11 (0.99)	.74	0.79	-0.04	0.07	.66
**7**	Rich countries taking advantage of poor countries	4.48 (0.80)	.70	0.75	-0.17	0.04	.63	4.66 (0.75)	.59	0.70	-0.14	0.22	.52	4.19 (0.98)	.70	0.76	-0.04	-0.01	.59
**8**	The dishonesty of rich countries	4.16 (1.02)	.65	0.76	0.10	0.04	.56	4.29 (0.94)	.58	0.69	0.02	0.09	.47	3.70 (1.13)	.73	0.84	0.14	0.01	.65
**9**	The political influences of rich countries	4.04 (1.32)	.57	0.70	0.16	0.00	.47	4.44 (0.81)	.53	0.63	0.01	-0.04	.40	4.10 (0.97)	.60	0.70	0.08	0.07	.48
**10**	The lack of talent and ability to succeed of poor countries	2.03 (1.37)	.72	-0.07	0.74	0.11	.66	2.31 (1.43)	.63	0.00	0.76	-0.01	.57	1.85 (1.13)	.53	0.00	0.70	-0.03	.48
**11**	The loose morals of poor countries	2.01 (1.14)	.64	0.08	0.78	0.00	.59	2.32 (1.36)	.60	0.07	0.71	0.07	.53	1.79 (1.03)	.53	0.06	0.66	0.10	.46
**12**	The laziness of poor countries	2.46 (1.20)	.69	0.02	0.78	0.08	.66	2.03 (1.23)	.61	-0.11	0.66	0.11	.55	1.56 (0.92)	.68	-0.03	0.84	-0.13	.68
**13**	The lack of effort at self-improvement of poor countries	2.25 (1.05)	.68	-0.13	0.74	0.04	.63	2.78 (1.40)	.71	0.05	0.83	0.00	.68	2.05 (1.07)	.72	-0.03	0.80	0.03	.67
**14**	The lack of thrift and proper money management of poor countries	2.37 (1.13)	.59	-0.08	0.62	-0.03	.40	3.63 (1.25)	.49	-0.01	0.69	-0.13	.42	2.58 (1.32)	.61	0.03	0.74	0.03	.55
**15**	The lack of personal drive and willingness to take risks of poor countries	1.09 (0.45)	.67	0.02	0.81	-0.04	.62	2.58 (1.39)	.71	-0.05	0.78	0.07	.67	1.87 (1.00)	.62	-0.10	0.60	0.28	.57
**16**	Some countries are just lucky and others are not	1.29 (1.13)	.62	-0.04	-0.11	0.87	.70	1.99 (1.20)	.50	0.02	0.10	0.64	.47	2.10 (1.16)	.52	0.08	-0.03	0.81	.66
**17**	It is god’s will	2.02 (1.11)	.44	0.10	0.21	0.56	.45	1.58 (1.13)	.42	-0.02	-0.11	0.74	.49	1.27 (0.75)	.35	-0.11	0.17	0.46	.30
**18**	That is what fate/destiny has in store for each of us	4.01 (1.11)	.63	-0.03	0.15	0.72	.65	1.86 (1.23)	.64	-0.02	0.05	0.81	.70	1.58 (0.98)	.48	-0.05	0.17	0.62	.47
**19**	The good or bad luck the country has compared to other countries	3.31 (1.14)	.64	0.06	0.12	0.68	.54	2.05 (1.24)	.57	0.00	0.18	0.66	.58	2.31 (1.15)	.59	-0.01	-0.05	0.85	.70
***Proportion of Explained Variance***				0.27	0.21	0.11				0.24	0.19	0.12				0.29	0.19	0.11	
***Cumulative Proportion of Explained Variance***				0.27	0.47	0.58				0.24	0.43	0.54				0.29	0.49	0.60	
***Cronbach’s α***				.86	.86	.72				.86	.84	.74				.91	.83	.69	
***M* (*SD*)**				4.18 (0.66)	1.91 (0.81)	1.59 (0.66)				4.51 (0.56)	2.61 (1.01)	1.87 (0.90)				4.08 (0.77)	1.95 (0.80)	1.81 (0.74)	

*Notes*. CICT = Corrected Item-Total Correlation; Com = Item communality.

The factors correlate with each other in a meaningful way. In all countries, the “rich countries” factor correlates negatively and in similar magnitude with the “poor countries” factor (*r*_*ITA*_ = -.25, *p* < .001; *r*_*RSA*_ = -.16, *p* = .013; *r*_*UK*_ = -.29, *p* < .001), and the “poor countries” is associated positively with the “fate” factor (*r*_*ITA*_ = .51, *p* < .001; *r*_*RSA*_ = .50, *p* < .001; *r*_*UK*_ = .38, *p* < .001). As for the relationship between “rich countries” and “fate” factors, we found a negative correlation in Italy (*r* = -.13, *p* = .042) and South Africa (*r* = -.15, *p* = .028) but not in the UK (*r* = .03, *p* = .604).

#### Correlational patterns

Tables in the (S2–S4 Tables) present the correlations between all the measured variables in all countries. Here, we discuss the most theoretically relevant links [87,117]. Specifically, we examined the correlations between the ACIS factor scores, their potential ideological underpinnings, and people’s beliefs about cross-country economic inequality and its remedies.

As illustrated in Table 2, the correlational pattern of “rich countries” and “poor countries” factors was overall consistent across countries and their correlations were complementary and of medium-to-strong size with respect to ideological orientation and attitudes toward cross-country inequality and its remedies. Specifically, the “rich countries” (vs. “poor countries”) factor was negatively (vs. positively) linked to beliefs in a just economic system, in social dominance, in meritocracy (but not for South African respondents), and in conservative political values (but not for South African respondents). Opposingly, evaluations of inequality as too large, immoral, unfair, and outrageous (but not for South African respondents) were positively linked to “rich countries” and negatively to “poor countries” factors. Similar correlations were found for support for labor migration and support for redistributive policies (except for South African respondents). National identification for British and Italian respondents was associated with lower levels of rich blaming and a higher level of poor blaming. These links were absent among South African respondents.

**Table 2:** Correlations between ACIS factors and relevant constructs in the two studies.

		Study 1			Study 2		
Factor	Variable	Italy (N = 246)	South Africa (N = 228)	UK (N = 248)	Italy (N = 239)	South Africa (N = 248)	UK (N = 249)
Rich countries	Perception of inequality	.40**	.38**	.54**	.38**	.43**	.48**
	Redistribution	.35**	.28**	.44**	.25**	.40**	.39**
	Migration	.27**	.39**	.36**	.34**	.35**	.42**
	Fairness	-.38**	-.22**	-.49**	-.36**	-.37**	-.50**
	Moralization	.47**	.35**	.47**	.38**	.48**	.47**
	Moral outrage	.39**	.39**	.50**	.35**	.57**	.47**
	Meritocracy	-.24**	.05	-.29**	-.26**	-.15*	-.29**
	Social dominance	-.44**	-.34**	-.45**	-.45**	-.41**	-.44**
	Economic system justification	-.53**	-.38**	-.50**	-.52**	-.37**	-.54**
	Political orientation	-.27**	-.05	-.47**	-.26**	-.17**	-.48**
	National identity	-.19**	.09	-.27**	-	-	-
	Identification with country	-	-	-	-.02	.14*	-.36**
	Identification with world	-	-	-	.01	-.12	.02
	Need for institutions	-	-	-	.18**	.16**	.36**
	Trust in institutions	-	-	-	-.16*	-.26**	.04
	Zero sum beliefs	-	-	-	.51**	.57**	.63**
Poor countries	Perception of inequality	-.37**	-.17*	-.34**	-.25**	-.08	-.42**
	Redistribution	-.27**	-.08	-.20**	-.17**	-.13*	-.33**
	Migration	-.27**	-.13*	-.18**	-.29**	-.06	-.44**
	Fairness	.30**	.15*	.35**	.18**	.30**	.40**
	Moralization	-.32**	-.16*	-.36**	-.27**	-.21**	-.35**
	Moral outrage	-.32**	-.07	-.34**	-.22**	-.24**	-.39**
	Meritocracy	.45**	.33**	.53**	.41**	.45**	.48**
	Social dominance	.48**	.23**	.38**	.42**	.19**	.41**
	Economic system justification	.49**	.35**	.52**	.55**	.43**	.58**
	Political orientation	.43**	.15*	.38**	.39**	.10	.45**
	National identity	.21**	.001	.30**	-	-	-
	Identification with country	-	-	-	.09	-.12	.**32**
	Identification with world	-	-	-	-.02	.13*	-.21**
	Need for institutions	-	-	-	-.23**	.05	-.29**
	Trust in institutions	-	-	-	-.09	.14*	-.24**
	Zero sum beliefs	-	-	-	-.15*	-.13*	-.23**
Fate	Perception of inequality	-.27**	-.15*	-.01	-.24**	-.07	-.15*
	Redistribution	-.08	.08	.07	-.11	.12	-.16*
	Migration	-.00	-.20**	-.04	-.11	.05	-.27**
	Fairness	.24**	.19**	.01	.16*	.08	.21**
	Moralization	-.19**	-.19**	-.07	-.23**	-.06	-.20**
	Moral outrage	-.15*	-.03	-.05	-.30**	.03	-.15*
	Meritocracy	.30**	.15*	.22**	.26**	.16*	.19**
	Social dominance	.41**	.17*	.17**	.32**	.26**	.24**
	Economic system justification	.35**	.30**	.26**	.45**	.36**	.28
	Political orientation	.16*	.11	.20**	.27**	.10	.23**
	National identity	.22**	-.03	.22**	-	-	-
	Identification with country	-	-	-	-.06	-.14*	.21**
	Identification with world	-	-	-	-.12	-.15*	-.08
	Need for institutions	-	-	-	-.15*	.11	-.09
	Trust in institutions	-	-	-	-.01	.11	-.12
	Zero sum beliefs	-	-	-	-.15*	.17**	-.11

*Note*. ** p < .001

* p < .05.

The “fate” factor showed a less consistent correlation pattern across countries and the correlations were generally weaker compared to the other two factors. Similarly to the “poor countries” factor, it related negatively to attitudes toward cross-country inequality and its remedies (except for British respondents, for whom no correlation was significant), and positively to ideological orientations. Further, the “fate” factor related positively to sense of national identity (except for South African respondents). Regression analysis (S5 Table) also indicated the less consistent relationships of this factor to cross-country inequality beliefs compared to the other two factors.

Regression analysis also indicated that adding sociodemographic variables (i.e., gender and age) does not alter the predictive efficacy of causal attributions on beliefs about cross-country economic inequality and its remedies.

### Discussion

Study 1 provides initial evidence for a three-factorial structure of the ACIS, its reliability, and criterion validity. The EFA revealed that the factors explained a satisfactory amount of variance [118], and reliability analysis indicated good internal consistency. The correlational pattern of “rich countries” and “poor countries” factors was overall complementary in relation to both ideological orientations and attitudes toward cross-country inequality. Concerning the correlational pattern of the “fate” factor, we found that it showed a similar pattern to the “poor countries” factor. Compared to it, “fate” factor correlations and regression coefficients were less consistent across countries and weaker.

Interestingly, we observed that South African respondents diverged from the Italian and British respondents with respect to some results. Such differences may best be explained by taking an intergroup perspective, where belonging to high- or low-income countries may prove relevant for the perception and explanation of inequality (intergroup causal attribution), and, more generally, for the development of cognitive, affective, and behavioral habits that reflect different levels of opportunity, status, and resources [119]. The cultural and normative climate of a given country may therefore shape how economic inequality is explained and which strategies are deemed useful to manage it. From this perspective, it is not surprising that conservatism and meritocracy beliefs were predictive of blaming the poor countries among UK and Italian, but not among South African participants. By the same token, British and Italian participants who strongly identified with their own (rich) nation held poor countries more accountable for international income gaps; this was not true for participants coming from a relatively poor and highly unequal country, namely South Africa. To establish the stability of the correlation pattern, the same relationships need to be examined independently. In Study 2, besides confirming the factorial structure of the ACIS and evaluating cross-country invariance, we aimed at testing the consistency of the correlations that emerged and provide support for the criterion validity of the ACIS.

## Study 2

In Study 1, we observed a general pattern that links blaming poor countries or fate (vs. blaming rich countries) with appraisals of cross-country inequality as justified, not to be contested, in line with a meritocratic and conservative worldview, and where differences between groups are inevitable to keep the world organized or in order. However, Study 1 overlooked more cognitive appraisals of inequality, particularly in relation to the evaluation of resources as a finite amount. Zero-sum beliefs represent the competitive view that one person’s gain will be another person’s loss and vice versa [120]. It is associated with political orientation [121] and people’s feeling that they are being taken advantage of and that the social system is illegitimate and unjust [122]. Thus, in Study 2, we will evaluate the relationship between cross-country inequality explanations and zero-sum beliefs.

Additionally, at the domestic level, economic inequality within a society relates to the trust people place in national institutions [123,124]. Institutional trust is an individual’s expectation that an institution will produce positive outcomes [125]. It is a crucial factor in democratic systems and a condition that increases support for welfare policies, especially when considered in conjunction with lay explanations for domestic poverty. Individuals who blame society for poverty may also have less trust in the society’s ability to resolve the problem. This might also happen for international institutions (e.g., the United Nations, the World Health Organization, and the International Monetary Fund) that facilitate global cooperation and strengthen relationships between countries by mediating conflicts and preventing less-wealthy countries from experiencing economic crises and hardship. Thus, in Study 2, we will evaluate the relationship between explanations for cross-country inequality and trust in international institutions. Furthermore, we will also explore their associations with the perceived need for these institutions.

### Sample and procedure

This study was approved by the Ethical Committee of the University of Padova (protocol number 4558). All participants provided informed written consent prior to study enrollment. We did not have access to information that could identify individual participants during or after data collection.

We recruited three Prolific samples from the same countries included in Study 1 (i.e., Italy, South Africa, and the UK) for a total of 784 participants. Specifically, 267 responses were collected from Italy, 263 from South Africa, and 254 from the UK. Those who participated in the first study were not allowed to participate in the second study, and workers were paid £1.50 for participating. Data were collected from April 26th to April 27th, 2022, two months after Study 1. The same questionnaire was administered in all countries. The questionnaire was written in English and took 13 minutes on average to complete.

After removing participants who did not meet inclusion criteria (i.e., not fluent in English, *n* = 14; not a citizen in the relevant country, *n* = 1; did not finish the questionnaire, *n* = 14; failed one or more of the four attentional checks, *n* = 19), the sample was composed of 736 participants: 239 from Italy, 248 from South Africa, and 249 from the UK. Sample sizes are considered adequate to confirm the factorial structure of the ACIS [126,127]. Furthermore, a sensitivity analysis run on G*Power 2 [100] showed that our smallest sample (N = 239) could detect two-tailed, bivariate correlations of r = |.13| with .80 power and α = .05. The descriptive statistics of the three samples are shown in S6 Table.

### Measures

The questionnaire was structured into three sections. In the first section, participants provided informed consent to participate in the study. In the second section, participants answered items investigating perceptions, causal attributions, and judgments of cross-country inequality. Differently from Study 1, we did not include items related to domestic inequality. In the third section, as in Study 1, participants answered ideology measures (i.e., social dominance, meritocracy, system justification, political orientation); further, socioeconomic (e.g., SSES, own country’s perceived economic position) and sociodemographic (e.g., gender, age, education) information was collected. Differently from Study 1, we measured national identity with the Inclusion of Other in the Self (IOS) scale [128], which assesses a similar construct and eases comparison across countries. We adapted the IOS scale to also measure individuals’ identification with the world population. Additionally, the following measures were added to the questionnaire.

#### Zero-sum beliefs

We measured how strongly participants believed that cross-country inequality is a zero-sum situation by asking them to rate 3 items (e.g., “If some countries get richer, it means that other countries get poorer”) on a 5-point scale (1 = *strongly disagree*, 5 = *strongly agree*). The items showed high internal consistency in all three countries (α_ITA_ = .80; α_UK_ = .87; α_RSA_ = .86). We computed the average of the answers, and higher scores indicated higher zero-sum beliefs.

#### Perceived need for, and trust in, international institutions

We measured how strongly participants think the world needs international institutions by asking them to rate a single-item question (i.e., “Do you think that we need international institutions (institutions that help governments to collaborate on important issues)?”) on a 5-point scale (1 = *not at all*, 5 = *absolutely*). Further, we assessed how much participants trust international institutions by asking them to rate four institutions (i.e., the United Nations, the World Health Organization, the International Monetary Fund, and the World Bank) on a 5-point scale (1 = *not at all*, 5 = *extremely*) plus an additional response option (6 = *I don’t know this institution*). We computed the average of the answers and higher scores indicated higher trust in international institutions.

#### Economic mobility of countries

Perception of individual social mobility is a core underpinning of economic inequality as it relates to the way people explain it. For instance, people who experience improvements in living standards through social mobility are more likely to attribute their success to dispositional characteristics [47]. In this study, we considered country social mobility, which refers to the belief that countries can change their economic position in the world hierarchy. Participants were asked to rate three items (i.e., “Rich countries can hardly become poor,” “A country can easily change its economic position in the world,” and “Poor countries can hardly become rich”) on a 5-point scale (1 = *strongly disagree*, 5 = *strongly agree*). Since internal consistency of the items was low in all countries (α_ITA_ = .45; α_UK_ = .56; α_RSA_ = .49), this scale was not further considered.

### Results

#### Confirmatory factor analysis

We analyzed the three-factor structure of the final ACIS in the three countries, using Maximum Likelihood (CFA). To assess the fit of the measurement model [129], we used the Comparative Fit Index (CFI), the Tuker-Lewis Index (TLI), the Root Mean Square Error of Approximation (RMSEA), and the Root Mean-Square Residual (RMSR). Model fit was judged using the following cutoff values: For the CFI and TLI, the fit was considered adequate if their values were larger than .90 [130], while values smaller than 0.10 and 0.08 suggested good model fit for RMSEA and RMSR, respectively [131]. The initial CFA evidenced that the item “It’s God’s will” underperformed in Italy (factor loading equal to .20) and in the UK (factor loading equal to .23). Thus, we decided to drop the item from the “fate” factor. We performed the CFA on the remaining 18 items, and the results confirmed the measurement model in the three countries (Table 3, lower panel). As shown in Table 3, all factor loadings are higher than .45 [113], except for the item “The loose morals of poor countries” in the South African sample and the item “That is what fate/destiny has in store for each of us” in the British sample, where factor loadings are slightly lower than the threshold (.42 and .37, respectively). We decided to retain these items as they show to be consistent in the other samples, and the overall model fit indices were satisfactory. The three factors correlated with each other in similar ways as they did in Study 1. The “rich countries” factor correlated negatively, and in similar magnitude, with the “poor countries” factor (-.26 < *r* < -.47, *p* < .001). The latter was associated positively with the “fate” factor (.25 < *r* < .37, *p* < .001). As for the relationship between the “rich countries” and “fate” factors, we found a negative correlation in Italy and the UK (-.15 < *r* < -.26, *p* < .023), but not in South Africa (*r* = -.01, *p* = .865).

**Table 3:** CFAs of the 18 ACIS items: Descriptive statistics, reliability, and factor loadings (Study 2).

Country	Factor	Item	Mean (SD)	CICT	Factor Loading	SE	*p*	95%CI
**UK**	**Rich countries**	1	4.08 (0.88)	.74	0.776	0.028	< .001	[0.721, 0.831]
		2	3.95 (0.96)	.72	0.753	0.030	< .001	[0.694, 0.812]
		3	4.00 (0.94)	.76	0.795	0.026	< .001	[0.744, 0.846]
		4	4.14 (0.96)	.74	0.778	0.028	< .001	[0.724, 0.833]
		5	3.98 (0.96)	.76	0.790	0.027	< .001	[0.738, 0.842]
		6	3.96 (0.97)	.75	0.775	0.028	< .001	[0.719, 0.830]
		7	3.59 (1.03)	.71	0.733	0.032	< .001	[0.671, 0.796]
		8	3.81 (0.99)	.69	0.710	0.034	< .001	[0.644, 0.777]
		9	4.11 (0.84)	.72	0.759	0.030	< .001	[0.701, 0.817]
	**Poor countries**	10	1.99 (1.12)	.67	0.730	0.035	< .001	[0.661, 0.799]
		11	2.00 (1.15)	.53	0.569	0.048	< .001	[0.475, 0.662]
		12	1.62 (0.82)	.66	0.739	0.035	< .001	[0.671, 0.806]
		13	2.19 (1.14)	.76	0.840	0.026	< .001	[0.788, 0.892]
		14	2.74 (1.20)	.53	0.569	0.048	< .001	[0.475, 0.663]
		15	1.95 (1.02)	.60	0.700	0.038	< .001	[0.626, 0.774]
	**Fate**	16	2.36 (1.11)	.59	0.788	0.070	< .001	[0.651, 0.924]
		18	1.64 (0.90)	.31	0.371	0.064	< .001	[0.245, 0.496]
** **		19	2.44 (1.12)	.43	0.783	0.069	< .001	[0.646, 0.919]
**Italy**	**Rich countries**	1	4.26 (0.81)	.69	0.741	0.034	< .001	[0.675, 0.807]
		2	3.99 (0.93)	.61	0.638	0.042	< .001	[0.555, 0.721]
		3	4.41 (0.74)	.68	0.714	0.036	< .001	[0.644, 0.784]
		4	4.45 (0.78)	.63	0.714	0.036	< .001	[0.644, 0.785]
		5	4.07 (0.93)	.56	0.587	0.046	< .001	[0.496, 0.678]
		6	4.42 (0.77)	.71	0.774	0.031	< .001	[0.714, 0.834]
		7	3.80 (0.93)	.62	0.636	0.042	< .001	[0.553, 0.720]
		8	4.28 (0.83)	.66	0.717	0.036	< .001	[0.647, 0.787]
		9	4.20 (0.78)	.59	0.606	0.045	< .001	[0.518, 0.694]
	**Poor countries**	10	1.75 (0.97)	.61	0.726	0.037	< .001	[0.654, 0.797]
		11	1.79 (0.99)	.57	0.634	0.044	< .001	[0.548, 0.720]
		12	1.52 (0.81)	.71	0.816	0.029	< .001	[0.759, 0.873]
		13	2.04 (1.08)	.70	0.773	0.032	< .001	[0.710, 0.837]
		14	2.92 (1.13)	.43	0.451	0.056	< .001	[0.341, 0.562]
		15	1.95 (1.06)	.59	0.653	0.043	< .001	[0.569, 0.736]
	**Fate**	16	1.98 (1.09)	.58	0.695	0.055	< .001	[0.588, 0.802]
		18	1.33 (0.73)	.42	0.523	0.060	< .001	[0.406, 0.640]
** **		19	2.10 (1.08)	.57	0.772	0.054	< .001	[0.667, 0.878]
**South Africa**	**Rich countries**	1	4.33 (0.87)	.67	0.698	0.035	< .001	[0.629, 0.767]
		2	4.39 (0.84)	.64	0.655	0.039	< .001	[0.578, 0.731]
		3	4.38 (0.95)	.73	0.749	0.031	< .001	[0.688, 0.809]
		4	4.59 (0.80)	.76	0.811	0.025	< .001	[0.762, 0.860]
		5	4.38 (0.93)	.75	0.778	0.028	< .001	[0.722, 0.833]
		6	4.47 (0.88)	.76	0.821	0.024	< .001	[0.775, 0.868]
		7	4.10 (1.08)	.69	0.718	0.034	< .001	[0.653, 0.784]
		8	4.52 (0.90)	.74	0.796	0.026	< .001	[0.744, 0.848]
		9	4.46 (0.81)	.61	0.630	0.041	< .001	[0.549, 0.710]
	**Poor countries**	10	2.31 (1.33)	.45	0.530	0.052	< .001	[0.428, 0.632]
		11	2.64 (1.41)	.39	0.423	0.058	< .001	[0.309, 0.537]
		12	2.30 (1.37)	.66	0.762	0.035	< .001	[0.693, 0.831]
		13	3.21 (1.31)	.66	0.754	0.036	< .001	[0.684, 0.824]
		14	4.03 (1.12)	.45	0.479	0.055	< .001	[0.371, 0.587]
		15	2.92 (1.43)	.64	0.763	0.035	< .001	[0.693, 0.832]
	**Fate**	16	2.06 (1.25)	.50	0.624	0.057	< .001	[0.513, 0.735]
		18	1.83 (1.10)	.52	0.686	0.055	< .001	[0.578, 0.794]
** **		19	2.06 (1.24)	.50	0.656	0.056	< .001	[0.547, 0.765]

**Model fit**	**TLI**	**CFI**	**RMSEA**	**90%CI RMSEA**	**SRMR**
UK	0.904	0.917	0.075	0.064; 0.085	0.075
Italy	0.921	0.932	0.060	0.048; 0.072	0.061
South Africa	0.940	0.948	0.054	0.041; 0.066	0.056

#### Multi-group confirmatory factor analysis

To determine whether the ACIS elicits similar responses in the sampled countries, we conducted a Multigroup-CFA (MG-CFA) [132], a covariance-based modeling technique that tests for the observed heterogeneity in a measurement model, or measurement invariance. MG-CFA hierarchically tests multiple levels of equivalence of model parameters (i.e., factor loadings, intercepts, and residuals) across countries. The first step is configural invariance, a model that tests whether the items load on the same latent factor. A RMSEA smaller than .08 is a good indicator of configural invariance [133]. The second step is metric invariance, a model in which the factor loadings are forced to be equal across groups. If metric invariance is met, regression coefficients can be safely compared across groups. The third step is the scalar invariance, a model that constrains the item intercepts to be equal across groups. It tests whether there is a uniform item bias present in an item. Scalar invariance is desirable because it enables mean comparisons across groups. Metric and scalar invariances are reached if the change in CFI and RMSEA between nested models is smaller than .01 and .015, respectively [134]. As shown in Table 4, we found support for configural and metric invariances but not for scalar invariance. Thus, while we must be cautious in comparing the means of the ACIS factors across countries, we can safely contrast their regression coefficients.

**Table 4:** ACIS measurement invariance: Fit indices from the multigroup CFA.

Type of Invariance	CFI	RMSEA	ΔCFI	ΔRMSEA
Configural invariance	0.932	0.064		
Metric invariance	0.923	0.065	0.009	0.002
Scalar invariance	0.889	0.075	0.033	0.010

#### Reliability

In this second examination, the three factors showed satisfactory internal consistency in all countries [116]. The Cronbach’s alpha for the “rich countries” factor was .88, .93, and .92, respectively for Italy, the UK, and South Africa. For the “poor countries” factor, it was equal to .83, .84, and .79, and for the “fate” factor it was .69, .67, and .69. Also, the corrected item-total correlations were larger than .30 in all countries [135], indicating coherence between any item and the other items composing the same factor.

#### Correlational patterns

We evaluated the correlations between the three ACIS factors scores and the other measured constructs. All correlations are reported in S7–S9 Tables, respectively for the Italian, South African, and British samples. As in Study 1, here we discuss the most theoretically relevant correlations, which are reported in Table 2.

Overall, the correlational pattern between ACIS factors, ideological orientations, and attitudes toward cross-country inequality and its remedies closely resembled the one of Study 1. The correlations of the “rich countries” and “poor countries” factors were once again complementary and of medium-to-strong sizes. Specifically, we found that the “rich countries” (vs. “poor countries”) factor correlated negatively (vs. positively) with the ideological measures and positively (vs. negatively) with attitudes toward cross-country inequality and its remedies. The pattern was consistent in all three countries. Also in line with Study 1, the “fate” factor showed a less consistent pattern across countries, correlations were weaker compared to the other two factors, and regression analysis (S10 Table) further support this last results. As the “poor countries” factor, it correlated positively with ideological orientations and negatively to attitudes toward inequality and its remedies. Different from Study 1, the correlation with evaluations of inequality emerged in Italy and the UK, but not in South Africa.

Similar to Study 1, regression analysis further indicated that the inclusion of sociodemographic variables (i.e., gender and age), does not modify the predictive effectiveness of causal attributions concerning beliefs about cross-country economic inequality and potential remedies.

In Study 2, we found a complementary correlation pattern between the “rich countries” and the “poor countries” factors with respect to zero-sum beliefs and the perceived need for international institutions, and these correlations were for the most part stable across countries. Specifically, the more people blamed rich countries (vs. poor countries) for inequality, the more (vs. less) they believed inequality is a zero-sum game and the more (vs. less) they perceived the need for international institutions.

Concerning trust in international institutions, we did not find a robust complementary pattern between the “rich countries” and “poor countries” factors across countries. Indeed, while the former correlated negatively with trust in all three samples, the latter correlated positively with trust in South Africa and negatively in the UK. Further, this correlation was not significant in the Italian sample. Thus, overall correlations between causal attributions and trust in international institutions were somewhat incoherent.

As for self-identification measures, stronger national identification related negatively to the “rich countries” factor and positively with the “poor countries” and “fate” factors in the British sample. The opposite pattern was found in the South African sample, for which national identity related positively to the “rich countries” factor, negatively to the “fate” factor, and not significantly to the “poor countries” factor. Italian national identity did not relate to explanations for cross-country inequality.

In contrast, identification with the world at large was unrelated to blaming rich countries for cross-country inequality but it was negatively associated with blaming poor countries in the UK and Italy, and positively in South Africa. For South African respondents, those who identified more with the world at large also endorsed less fatalistic explanations.

### Discussion

Study 2 provides additional support for the three-factorial structure of the ACIS. The CFA confirmed the measurement model, and the Multigroup CFA supported its configural and metric invariances across the investigated countries. This allows us to safely compare correlation coefficients across samples and to gain initial evidence of the factorial validity of the ACIS. Reliability analysis further supported the good internal consistency of the scale. The correlational patterns of “rich countries” and “poor countries” factors with ideological orientations and evaluations of cross-country inequality were overall similar to that of Study 1. This is of importance as it supports the criterion validity of the two factors. Causal attributions for cross-country inequality were linked to the ideological orientations and attitudes toward cross–country inequality in a meaningful and largely coherent way across countries. For instance, the greater the participants’ social dominance orientation and the more they justified the system, the less they held the rich, and the more they held the poor countries responsible for international inequality. Also, the more participants blamed the rich countries for current levels of economic inequality, the more they perceived inequality as too large, immoral, unfair, and outrageous. Further, for all three countries, zero-sum beliefs were positively related to blaming the rich rather than the poor countries for the current state of affairs.

At the same time, there were also clear distinctions between the three countries that are consistent with an intergroup perspective and that seem to reflect the relative status of each country in the international hierarchy. Among the three countries considered here, the UK is the one that is highest ranked in the 2023 international hierarchy on various indices such as the pro-capita GDP, Human Development Index, and the Military Strength Index. Not surprisingly then, the greater the identification with the nation, the more British participants attributed economic inequality to the poor, and the less to the rich countries. To the contrary, participants from South Africa (who rank lowest in the international hierarchy) showed an opposite pattern. The more they identified with their country, the more they blamed inequality on the rich (but not on the poor) countries. Italy occupied an intermediate position, showing no systematic correlations between national identification and causal attributions. This points to an interesting self-serving bias that varies in function of the relative standing of one’s country in the global hierarchy.

As in Study 1, the correlations involving the “fate” factor were similar to the “poor countries” factor. Again, the pattern we observed for the former is less consistent than for the latter. Also, it showed lower factor loading in the CFA and reliability compared to the other two factors. However, the greater instability of the fatalistic factor is not particularly new in the literature on causal attributions for inequality, as this factor did not emerge consistently across various studies [87,102,136,137]. Possibly, the inconsistency of this factor might be explained by a strong relationship with one’s religiosity, not investigated here. Religion strongly impacts how people experience and interpret reality around them [138,139].

Overall, the correlation analyses suggest that lay explanations of cross-country inequality are a psychological reality intertwined with various personal beliefs about cross-country inequality itself and relevant ideological orientations.

## General discussion

Psychological literature has consistently shown that the causal explanations people provide to economic phenomena play a key role in shaping people’s opinions and attitudes and directing our behavior [29,30,140–143]. Our research has shown that this occurs not only when people explain phenomena in close proximity to the person, such as domestic poverty and economic success, but also when they explain global phenomena, such as cross-country economic inequality. So far, only a few studies have investigated cross-country inequality from a psychological perspective, yielding relevant knowledge about how this phenomenon is perceived by people [83–85]. Even fewer studies have investigated the lay theories people employ to explain this phenomenon [70] In this paper, we investigated causal attributions for cross-country inequality through two studies involving independent samples from three different countries (i.e., South Africa, Italy, and the UK). We developed and validated a scale that measures the endorsement of such attributions (i.e., the ACIS). Importantly, we also investigated how these attributions link to judgments about cross-country inequality, its solutions, and people’s ideology.

Exploratory (Study 1) and confirmatory (Study 2) factor analyses, reliability analyses, and correlational patterns confirm that this 18-item scale reliably measures three types of causal attributions, the structure of which replicates in all the investigated countries: blame attributed to “rich countries,” “poor countries,” and “fate.” Considering the content of the items that make up the factors, we can see that they are conceptually similar but not completely overlapping with those that have been identified in research on the causal attributions for domestic poverty and wealth [87,138], namely situational, dispositional, and fatalistic causes. In fact, the “rich countries” factor emphasizes not only the relevance of the systematic economic advantage of rich over poor countries based on situational factors (e.g., “Economic systems that create advantages for rich countries”), but also characteristics of these countries (e.g., greediness) that resemble individuals’ dispositional features. It is interesting to note that when it comes to rich countries, situational and dispositional aspects go together. This might be due to the fact that international economic and political systems are perceived to be driven by rich countries and their maliciousness. Rich countries play a major role in determining the worldwide quality of life, especially in developing countries. They fuel global climate change [144], restrict mobility to citizens from developing countries (i.e., global mobility divide; [145]), externalize environmental costs of production and consumption (e.g., hazardous waste trade; [146]), and exploit resources and cheap labor forces from developing countries [147]. The factor “poor countries,” instead, focuses on the responsibilities of poor countries in determining their economic disadvantage (e.g., “The lack of personal drive and willingness to take risks of poor countries”), and no global, systematic element appears. Finally, the factor “fate” captures beliefs related to fate and luck (e.g., “Some countries are just lucky and others are not”). Therefore, the current studies demonstrate the good psychometric properties of ACIS and suggest interesting theoretical insights, such as that when it comes to inequality, the distinction between the three targets to blame (i.e., poor, rich, fate) is more important than that between situational and dispositional attributions.

We can advance different explanations for why lay theories of cross-country economic inequality diverge in some respects from the lay theories of poverty (or, to a lesser degree of wealth) commonly found in the literature. First, one may speculate that when causally explaining inequality (rather than poverty), the focus shifts from the “how” to the “who.” By definition, inequalities are framed in terms of intergroup relations, and -as such- they clearly specify the groups that are involved in the comparison (rich vs. poor countries), whereas the assessment of poverty (or wealth) by itself may entail a focus on individual differences. Reasoning about intergroup inequality seems to focus on inferring responsibility and, therefore, on whom to blame [148]. This interpretation deserves further research as future studies directly comparing the assessment of the two levels may indeed provide insights into the causal attribution process behind these two issues.

The second interpretation rests on the high cognitive complexity that is needed to understand economic inequality, especially cross-country inequality. It might be a far too difficult task for laypeople to identify how rich and poor countries ended up in their relative positions on the economic ladder. The fact that causal inferences take a specific shape according to the complexity of the issue under consideration is in line with previous studies on environmental issues [149].

Third, as reviewed in the introduction, the literature investigating causal attributions for economic outcomes has focused primarily on lay theories that people use to explain domestic poverty or, to a lesser extent, domestic wealth. A few studies (e.g., [70]) have attempted to integrate both economic phenomena into a unified framework and, thus, to understand how people explain economic differences between rich and poor, both at domestic or cross-country levels. If one conceives economic inequality as an intergroup process, then Pettigrew’s Ultimate Attribution Error (UAE [73]; but see also [150]) may offer a useful lens to understand how lay people explain income gaps. The ultimate attribution error describes a general tendency among groups to be positively biased toward themselves (i.e., target-serving bias) and, in many cases, to be negatively biased toward persons in other groups (i.e., target-derogating bias). Although ingroup favoritism is highly prevalent in groups occupying symmetrical social positions, it does not always occur in cases of social asymmetry. Indeed, when social actors hold asymmetric positions, as in the case of rich and poor countries, ingroup favoritism may decrease or even be reversed. Studies that pointed in this direction [151,152] revealed how sensitive between-group attributions are to the relative position of the groups in question [150,153].

The patterns of correlations we observed in the two studies provided the first evidence for the utility of the ACIS by demonstrating medium-to-strong-sized correlations with criterion variables such as judgments about cross-country inequality (e.g., the perceived size of cross-country inequality, its unfairness, and immorality), attitudes toward some of its remedies (e.g., redistribution and work migration), and ideological orientation (e.g., economic system justification, meritocratic beliefs, and political orientation). This suggests that the ACIS is suitable to assess individual differences in beliefs. In addition, it is also interesting to observe the direction of these correlations. Indeed, the more strongly a person believes that inequality between countries is due to the systematic advantage of rich countries, the more likely they are to perceive inequality as unacceptable. In addition, they are also more likely to support greater taxation of rich countries (i.e., a form of international redistribution) and labor migration from poor countries. In contrast, arguing more strongly that cross-country inequality is to be attributed to the dispositional characteristics of poor countries is negatively associated with the same constructs. Analogous correlational patterns were found for ideology. In both studies those who blamed rich countries were less likely to adopt system-legitimizing beliefs (e.g., social dominance, meritocracy). These results contribute to the validity of the two dimensions since similar relationships emerge in the literature on causal attributions for domestic poverty and wealth.

Being the first of its kind, our research necessarily has both strengths and weaknesses. One of the main strengths of our work is that the ACIS was validated on samples from three different countries that vary in both wealth and economic inequality, though Italy and the UK are much more similar. Recent statistics show that Italy and the UK hold 2.5% and 3.5% of global wealth, and have a similar level of economic inequality, namely .352 and .326, respectively [86]. In contrast, South Africa holds only 0.22% of global wealth and is currently the most unequal country in the world with a Gini index of 63. Despite the evident geographical, cultural, and economic differences, the factorial structure of the ACIS was remarkably consistent across these samples. However, the samples were recruited through Prolific Academic, which employs a first-come, first-served convenience sampling method. Although the answers provided by Prolific respondents are qualitatively better than those provided by other respondents [154], and some demographic characteristics (i.e., age and gender) of our samples align with national benchmarks, future studies should establish the robustness of the factorial structure of the ACIS on representative samples of national populations. Further, the questionnaire was administered only in English. While this is the official language of the UK and one of the official languages in South Africa, it is not for Italy. Although our participants were screened based on English proficiency (see Method sections), future studies might consider testing all participants in their native language and, possibly, corroborating its psychometric characteristics. Indeed, it would be useful to employ a broadly cross-cultural approach to examine the factorial structure and nomological network of the ACIS factors in contexts that differ not only on economic development and Gini but also on human development (e.g., Human Development Index) and societal values (e.g., individualism-collectivism). A further limitation is that both studies have a correlational nature that prevents us from drawing conclusions about the underlying cause-effect relationships between the ACIS factors and the other measured constructs. Future experimental research may identify the conditions that favor the adherence to specific attributions and, by manipulating them, observe their psychological and behavioral effects (e.g., preferences for policy on foreign aid, climate change assistance, or prosocial behavior). Finally, we would like to acknowledge the role that social desirability bias may have played in our study. Individuals’ inclination to present themselves in a favorable light, conforming to prevailing social norms, may potentially compromise research findings and is particularly prevalent in research addressing sensitive social topics. To mitigate the impact of social desirability bias, we used self-administration and ensured participants’ anonymity, which are two well-known methods for bias prevention [155]. However, we acknowledge that this bias may have influenced participants’ reported agreement with the items related to "poor countries," which indeed were less endorsed compared to the "rich countries" factor. Nonetheless, we believe that our specific topic (i.e., cross-country economic inequality) may not be highly susceptible to this bias given that it is not a particularly sensitive or culturally sanctioned issue. Thus, it is unlikely to have impacted the correlations or the scale itself.

This paper contributes to the literature in several ways. From a methodological perspective, we provide scholars with a cross-culturally validated measure of causal attributions for cross-country inequality, which the literature currently lacks. Secondly, from a theoretical perspective, our results suggest that the causal attributions for cross-country inequality, conceived as an intergroup process, might differ from those underlying explanations of domestic poverty and wealth. Further, they show how people’s explanations to make sense of cross-country inequality link to relevant psychological constructs, such as beliefs about cross-country inequality and ideological orientations. We hope this scale will prove useful to researchers who aim to study people’s perceptions of economic inequality and their relationship to other constructs of interest.

## Supporting information

S1 Checklist STROBE statement—Checklist of items that should be included in reports of observational studies. (DOCX)

S1 Table Descriptive statistics of the samples and measures by country (Study 1). (DOCX)

S2 Table Correlations for the Italian sample (Study 1; n = 246). (DOCX)

S3 Table Correlations for the South African sample (Study 1; n = 228). (DOCX)

S4 Table Correlations for the British sample (Study 1; n = 248). (DOCX)

S5 Table Regression results of causal attributions on inequality appraisals, Study 1 (N = 722). (DOCX)

S6 Table Descriptive statistics of the samples and measures by country (Study 2). (DOCX)

S7 Table Correlations for the Italian sample (Study 2; n = 239). (DOCX)

S8 Table Correlations for the South African sample (Study 2; n = 248). (DOCX)

S9 Table Correlations for the British sample (Study 2; n = 249). (DOCX)

S10 Table Regression results of causal attributions on inequality appraisals, Study 2 (N = 736). (DOCX)
