# Supplementary material for: Why are some countries rich and others poor? development and validation of the attributions for Cross-Country Inequality Scale (ACIS)
Source: PLoS One. 2024 Feb 27;19(2):e0298222. doi: 10.1371/journal.pone.0298222 (PMC10898736; doi:10.1371/journal.pone.0298222)
Supplement: S1 Checklist — (DOCX) [file pone.0298222.s001.docx]

STROBE Statement—checklist of items that should be included in reports of observational studies

|  | **Item No.** | **Recommendation** | **Page  No.** | | **Relevant text from manuscript** |  |
| --- | --- | --- | --- | --- | --- | --- |
| **Title and abstract** | 1 | (*a*) Indicate the study’s design with a commonly used term in the title or the abstract | *2* | | *Accordingly, this paper aims to develop and validate with two cross-sectional studies the Attributions for Cross-Country Inequality Scale (ACIS), which assesses how people explain cross-country economic inequality* |  |
|  |  | (*b*) Provide in the abstract an informative and balanced summary of what was done and what was found | *2* | | *After selecting and adapting items from existing scales of attributions for poverty and wealth, in Study 1, we tested the factorial structure of this initial pool of items in three countries with different levels of economic development and inequality, namely, Italy (n = 246), the UK (n = 248), and South Africa (n = 228). Three causal dimensions emerged from the Exploratory Factor Analysis: “rich countries” (blaming the systematic advantage of and exploitation by rich countries), “poor countries” (blaming the dispositional inadequacy and faults of poor countries), and “fate” (blaming destiny and luck). The retained items were administered in Study 2 to three new samples from Italy (n = 239), the UK (n = 249), and South Africa (n = 248). Confirmatory Factor Analysis (CFA) corroborated the factorial structure of the ACIS, and Multi-Group CFA supported configural and metric invariances of the scale across countries. In addition, we show internal consistency and construct validity of the scale: the scale correlates with relevant constructs (e.g., beliefs about cross-country inequality and ideological orientation) and attitudes toward relevant policies related to international redistribution and migration. Overall, the scale is a valid instrument to assess causal attribution for cross-national inequality and is reliable across countries. By focusing on resource distribution from an international perspective, this scale will allow researchers to broaden the discussion on economic inequality to a global level.* |  |
| **Introduction** | | | |  | | |
| Background/rationale | 2 | Explain the scientific background and rationale for the investigation being reported | *3-5* | | *We live in a highly unequal world and this great difference in wealth and income between countries is self-evident: In rich countries, people are healthier and live longer, they are much better educated and have access to a range of amenities and options in life that are precluded to people in poor countries. [...]*  *As a result of globalization, we are all interconnected; problems such as poverty, climate change, and migration are never confined to one country alone, and even the richest countries have people living in poverty. Given the high levels of cross-country inequality and considering that it accounts for 32% of global economic inequality (i.e., inequality among all individuals on earth; [2]), reducing global inequality is a central point of the agenda of international organizations, such as the United Nations [12]. The public and policymakers should contemplate possible policy options (e.g., immigration and international aid policies) aimed at this and put them into practice. For these policies to be supported by the population, it is critical to investigate the lay theories through which people explain the economic gap between countries, which leads to the perception of what kind of social change is needed [13].*  *Understanding how people explain inequality is relevant because it is closely intertwined with the degree to which people tolerate inequality. For example, endorsing situational causes of poverty and wealth is related to individuals’ aversion to economic inequality [14], perceived lack of economic mobility [15], and the amount of inequality an individual perceives as fair [16] or as immoral and outraging [17,18]. Moreover, different causal explanations of poverty influence how people view the poor [19] and whether they believe economic resources should be shared with those who need them. [...]. As of now, however, the literature lacks an instrument to assess causal attributions for cross-country inequality. Thus, providing the scientific community with a new tool to measure how people make sense of cross-country economic inequality is useful and timely as it may advance our knowledge of how individuals interpret and react to global phenomena.* |  |
| Objectives | 3 | State specific objectives, including any prespecified hypotheses | *5* | | *This paper aims to develop and validate a new tool, the Attributions for Cross-Country Inequality Scale (ACIS), designed to measure individuals’ perceptions of the causes of economic inequality between nations. [...]*  *Below, we will review the literature and current measures of causal attributions for poverty and wealth to highlight the ways in which a scale such as ours would contribute to the literature on economic inequality: (a) by validating this new measure across low- and high-income countries; (b) by focusing on economic inequality rather than poverty and wealth; and (c) by providing evidence on the lay theories about cross-country, rather than domestic, inequality.* |  |
| **Methods** | | | |  | | |
| Study design | 4 | Present key elements of study design early in the paper | *5* | | *We first generated items using a top-down approach. Then, we tested the factorial structure of the scale through exploratory (Study 1) and confirmatory (Study 2) factor analytic approaches. We assessed criterion validity via correlational analyses between the ACIS factors and relevant socio-psychological constructs, such as judgments of and reactions to inequality (i.e., unfairness, outrage, and immorality) and ideological orientations (i.e., social dominance orientation, economic system justification, meritocratic beliefs, and political orientation).* |  |
| Setting | 5 | Describe the setting, locations, and relevant dates, including periods of recruitment, exposure, follow-up, and data collection | *10 (Study 1)*  *22 (Study 2)* | | *We developed Study 1 on Qualtrics (https://www.qualtrics.com) and distributed it to English-speaking Prolific Academic panelists from Italy, South Africa, and the UK. Prolific workers were paid £2.50 for participating in the study. Data collection took place from February 8th to February 14th, 2022.*  *We recruited three Prolific samples from the same countries included in Study 1 (i.e., Italy, South Africa, and the UK) for a total of 784 participants. Specifically, 267 responses were collected from Italy, 263 from South Africa, and 254 from the UK. Those who participated in the first study were not allowed to participate in the second study, and workers were paid £1.50 for participating. Data were collected from April 26th to April 27th, 2022, two months after Study 1.* |  |
| Participants | 6 | (*a*) *Cohort study*—Give the eligibility criteria, and the sources and methods of selection of participants. Describe methods of follow-up  *Case-control study*—Give the eligibility criteria, and the sources and methods of case ascertainment and control selection. Give the rationale for the choice of cases and controls  *Cross-sectional study*—Give the eligibility criteria, and the sources and methods of selection of participants | *10 (Study 1)*  *22 (Study 1)* | | *An initial sample of 828 participants was recruited. Specifically, 264 responses were collected from Italy, 290 from South Africa, and 274 from the UK.*  *After removing participants who did not meet inclusion criteria (i.e., access with a smartphone, n = 35; not fluent in English, n = 4; not a citizen or resident in the target country, n = 4; not pass at least one of the seven attentional checks, n = 41; not finished the questionnaire, n = 22), the final sample included 248 participants from the UK, 246 from Italy, and 228 from South Africa.*  *We recruited three Prolific samples from the same countries included in Study 1 (i.e., Italy, South Africa, and the UK) for a total of 784 participants. [...]*  *After removing participants who did not meet inclusion criteria (i.e., not fluent in English, n = 14; not a citizen in the relevant country, n = 1; did not finish the survey, n = 14; or not pass at least one of the four attentional checks, n = 19), the sample was composed of 736 participants: 239 from Italy, 248 from South Africa, and 249 from the UK.* |  |
|  |  | (*b*) *Cohort study*—For matched studies, give matching criteria and number of exposed and unexposed  *Case-control study*—For matched studies, give matching criteria and the number of controls per case | *N/A* | | *N/A* |  |
| Variables | 7 | Clearly define all outcomes, exposures, predictors, potential confounders, and effect modifiers. Give diagnostic criteria, if applicable | *10-13 (Study 1)*  *22-23 (Study 2)* | | *Databases, analysis scripts, and research material can be found online on OSF: https://osf.io/wbdh5/?view_only=4df676585024495dad0037cb6f0a3e7a* |  |
| Data sources/ measurement | 8* | For each variable of interest, give sources of data and details of methods of assessment (measurement). Describe comparability of assessment methods if there is more than one group | *10-13 (Study 1)*  *22-23 (Study 2)* | | *Databases, analysis scripts, and research material can be found online on OSF: https://osf.io/wbdh5/?view_only=4df676585024495dad0037cb6f0a3e7a* |  |
| Bias | 9 | Describe any efforts to address potential sources of bias | *10 (Study 1)*  *22 (Study 2)* | | *After removing participants who did not meet inclusion criteria (i.e., […]; not pass at least one of the seven attentional checks), the sample was composed of […].To further ensure the quality of the responses, we checked the geographic location from where the questionnaire was completed and we did not find inconsistencies.*  *After removing participants who did not meet inclusion criteria (i.e., […]; not pass at least one of the four attentional checks), the sample was composed of […].* |  |
| Study size | 10 | Explain how the study size was arrived at | *10 (Study 1)*  *22 (Study 2)* | | *Sample sizes were adequate to explore the factorial structure of the ACIS. As Kyriazos* [*[82]*](https://www.zotero.org/google-docs/?JV8ssn) *indicated, a sample size of at least 200 offers adequate statistical power for measures of up to 40 items* [*[83]*](https://www.zotero.org/google-docs/?Gap0ys)*.*  *Sample sizes are considered adequate to confirm the factorial structure of the ACIS [109,110].* |  |

| Quantitative variables | 11 | Explain how quantitative variables were handled in the analyses. If applicable, describe which groupings were chosen and why | *13 (Study 1)*  *23 (Study 1)* | *[Referring to ideological mesures] For all these measures we computed the average of the answers, and higher scores indicated higher meritocratic beliefs, system justification, and orientation to social dominance.*  *We measured how strongly participants believed that cross-country inequality is a zero-sum situation by asking them to rate 3 items [...] We computed the average of the answers, and higher scores indicated higher zero-sum beliefs. [...]*  *Further, we assessed how much participants trust international institutions by asking them to rate four institutions. [...] We computed the average of the answers and higher scores indicated higher trust in international institutions.* |
| --- | --- | --- | --- | --- |
| Statistical methods | 12 | (*a*) Describe all statistical methods, including those used to control for confounding | *13-17 (Study 1)*  *24-29 (Study 2)* | *To examine the factorial structure of the 38 causal attribution items in the three countries, we conducted EFAs (Maximum Likelihood) with oblique rotation (i.e., oblimin) given that the factors of the scales were not assumed to be orthogonal. […] Factors showed satisfactory internal consistency in all countries (see Table 1;* [*[98]*](https://www.zotero.org/google-docs/?C747xK)*). Also, the corrected item-total correlations was larger than .30 in all countries* [*[95]*](https://www.zotero.org/google-docs/?uarshT)*, indicating coherence between any item and the other items composing the same factor. […]Tables in the Supplementary Materials (Tables S2, S3, and S4) present the correlations between all the measured variables in all countries. […] Specifically, we examined the correlations between the ACIS factor scores, their potential ideological underpinnings, and people’s beliefs about cross-country economic inequality and its remedies.*  *We analyzed the three-factor structure of the final ACIS in the three countries, using Maximum Likelihood (CFA). To assess the fit of the measurement model* [*[112]*](https://www.zotero.org/google-docs/?ln2ECm)*, we used the Comparative Fit Index (CFI), the Tuker-Lewis Index (TLI), the Root Mean Square Error of Approximation (RMSEA), and the Root Mean-Square Residual (RMSR). Model fit was judged using the following cutoff values: For the CFI and TLI, the fit was considered adequate if their values were larger than .90* [*[113]*](https://www.zotero.org/google-docs/?PpUkMe)*, while values smaller than 0.10 and 0.08 suggested good model fit for RMSEA and RMSR, respectively* [*[114]*](https://www.zotero.org/google-docs/?OSKR3d)*. […]To determine whether the ACIS elicits similar responses in the sampled countries, we conducted a Multigroup-CFA (MG-CFA)* [*[115]*](https://www.zotero.org/google-docs/?TJyR8Z)*, a covariance-based modeling technique that tests for the observed heterogeneity in a measurement model, or measurement invariance. MG-CFA hierarchically tests multiple levels of equivalence of model parameters (i.e., factor loadings, intercepts, and residuals) across countries. […] In this second examination, the three factors showed satisfactory internal consistency in all countries* [*[98]*](https://www.zotero.org/google-docs/?zcTmoF)*. […] We evaluated the correlations between the three ACIS factors scores and the other measured constructs. All correlations are reported in Tables S6, S7, and S8, respectively for the Italian, South African, and British samples.* |
|  |  | (*b*) Describe any methods used to examine subgroups and interactions | *N/A* | *No subgroups and/or interactions were examined.* |
|  |  | (*c*) Explain how missing data were addressed | *N/A* | *No missing data were detected.* |
|  |  | (*d*) *Cohort study*—If applicable, explain how loss to follow-up was addressed  *Case-control study*—If applicable, explain how matching of cases and controls was addressed  *Cross-sectional study*—If applicable, describe analytical methods taking account of sampling strategy | *N/A* | *N/A* |
|  |  | (*e*) Describe any sensitivity analyses | *N/A* | *N/A* |
| **Results** | | | | |
| Participants | 13* | (a) Report numbers of individuals at each stage of study—eg numbers potentially eligible, examined for eligibility, confirmed eligible, included in the study, completing follow-up, and analysed | *11 (Study 1)*  *22 (Study 2)* | *An initial sample of 828 participants was recruited. Specifically, 264 responses were collected from Italy, 290 from South Africa, and 274 from the UK.*  *After removing participants who did not meet inclusion criteria (i.e., access with a smartphone, n = 35; not fluent in English, n = 4; not a citizen or resident in the target country, n = 4; not pass at least one of the seven attentional checks, n = 41; not finished the questionnaire, n = 22), the final sample included 248 participants from the UK, 246 from Italy, and 228 from South Africa.*  *We recruited three Prolific samples from the same countries included in Study 1 (i.e., Italy, South Africa, and the UK) for a total of 784 participants. Specifically, 267 responses were collected from Italy, 263 from South Africa, and 254 from the UK. […] After removing participants who did not meet inclusion criteria (i.e., not fluent in English, n = 14; not a citizen in the relevant country, n = 1; did not finish the survey, n = 14; or not pass at least one of the four attentional checks, n = 19), the sample was composed of 736 participants: 239 from Italy, 248 from South Africa, and 249 from the UK.* |
|  |  | (b) Give reasons for non-participation at each stage | *N/A* | *N/A* |
|  |  | (c) Consider use of a flow diagram | *N/A* | *N/A* |
| Descriptive data | 14* | (a) Give characteristics of study participants (eg demographic, clinical, social) and information on exposures and potential confounders | *10*  *22* | *Study 1) The descriptive statistics of the three samples are shown in Table S1.*  *Study 2) The descriptive statistics of the three samples are shown in Table S5.* |
|  |  | (b) Indicate number of participants with missing data for each variable of interest | *N/A* | *No missing data were detected.* |
|  |  | (c) *Cohort study*—Summarise follow-up time (eg, average and total amount) | *N/A* | *N/A* |
| Outcome data | 15* | *Cohort study*—Report numbers of outcome events or summary measures over time | *N/A* | *N/A* |
|  |  | *Case-control study—*Report numbers in each exposure category, or summary measures of exposure | *N/A* | *N/A* |
|  |  | *Cross-sectional study—*Report numbers of outcome events or summary measures | *N/A* | *N/A* |
| Main results | 16 | (*a*) Give unadjusted estimates and, if applicable, confounder-adjusted estimates and their precision (eg, 95% confidence interval). Make clear which confounders were adjusted for and why they were included | *N/A* | *N/A* |
|  |  | (*b*) Report category boundaries when continuous variables were categorized | *N/A* | *N/A* |
|  |  | (*c*) If relevant, consider translating estimates of relative risk into absolute risk for a meaningful time period | *N/A* | *N/A* |

| Other analyses | 17 | Report other analyses done—eg analyses of subgroups and interactions, and sensitivity analyses | *N/A* | *N/A* |
| --- | --- | --- | --- | --- |
| **Discussion** | | | | |
| Key results | 18 | Summarise key results with reference to study objectives | *31-32* | *In this paper, we investigated causal attributions for cross-country inequality through two studies involving independent samples from three different countries (i.e., South Africa, Italy, and the UK). We developed and validated a scale that measures the endorsement of such attributions (i.e., the ACIS). Importantly, we also investigated how these attributions link to judgments about cross-country inequality, its solutions, and people’s ideology.*  *Exploratory (Study 1) and confirmatory (Study 2) factor analyses, reliability analyses, and correlational patterns confirm that this 18-item scale reliably measures three types of causal attributions, the structure of which replicates in all the investigated countries: blame attributed to “rich countries,” “poor countries,” and “fate.”*  *[…] The patterns of correlations we observed in the two studies provided the first evidence for the utility of the ACIS by demonstrating medium-to-strong-sized correlations with criterion variables such as judgments about cross-country inequality (e.g., the perceived size of cross-country inequality, its unfairness, and immorality), attitudes toward some of its remedies (e.g., redistribution and work migration), and ideological orientation (e.g., economic system justification, meritocratic beliefs, and political orientation). This suggests that the ACIS is suitable to assess individual differences in beliefs. In addition, it is also interesting to observe the direction of these correlations.* |
| Limitations | 19 | Discuss limitations of the study, taking into account sources of potential bias or imprecision. Discuss both direction and magnitude of any potential bias | *34-35* | *Being the first of its kind, our research necessarily has both strengths and weaknesses. One of the main strengths of our work is that the ACIS was validated on samples from three different countries that vary in both wealth and economic inequality, though Italy and the UK are much more similar. […] However, the samples were recruited through Prolific Academic. Although the responses provided by Prolific respondents are qualitatively better than those provided by other respondents* [*[138]*](https://www.zotero.org/google-docs/?IOrUVA)*, future studies should establish the robustness of the factorial structure of the ACIS on representative samples. Further, the questionnaire was administered only in English. While this is the official language of the UK and one of the official languages in South Africa, it is not for Italy. Although our participants were screened based on English proficiency (see Method sections), future studies might consider testing all participants in their native language and, possibly, corroborating its psychometric characteristics. […] A further limitation is that both studies have a correlational nature that prevents us from drawing conclusions about the underlying cause-effect relationships between the ACIS factors and the other measured constructs.* |
| Interpretation | 20 | Give a cautious overall interpretation of results considering objectives, limitations, multiplicity of analyses, results from similar studies, and other relevant evidence | *33-34* | *We can advance different explanations for why lay theories of cross-country economic inequality diverge in some respects from the lay theories of poverty (or, to a lesser degree of wealth) commonly found in the literature. First, one may speculate that when causally explaining inequality (rather than poverty), the focus shifts from the “how” to the “who.” [...]. The second interpretation rests on the high cognitive complexity that is needed to understand economic inequality, especially cross-country inequality. [...]* |
| Generalisability | 21 | Discuss the generalisability (external validity) of the study results | *35-36* | *From a methodological perspective, we provide scholars with a cross-culturally validated measure of causal attributions for cross-country inequality, which the literature currently lacks. Secondly, from a theoretical perspective, our results suggest that the causal attributions for cross-country inequality, conceived as an intergroup process, might differ from those underlying explanations of domestic poverty and wealth. Further, they show how people’s explanations to make sense of cross-country inequality link to relevant psychological constructs, such as beliefs about cross-country inequality and ideological orientations. We hope this scale will prove useful to researchers who aim to study people’s perceptions of economic inequality and their relationship to other constructs of interest.* |
| **Other information** | |  | | |
| Funding | 22 | Give the source of funding and the role of the funders for the present study and, if applicable, for the original study on which the present article is based | *N/A* | *This information was provided at submission within the editorial manager system.* |

*Give information separately for cases and controls in case-control studies and, if applicable, for exposed and unexposed groups in cohort and cross-sectional studies.

**Note:** An Explanation and Elaboration article discusses each checklist item and gives methodological background and published examples of transparent reporting. The STROBE checklist is best used in conjunction with this article (freely available on the Web sites of PLoS Medicine at http://www.plosmedicine.org/, Annals of Internal Medicine at http://www.annals.org/, and Epidemiology at http://www.epidem.com/). Information on the STROBE Initiative is available at www.strobe-statement.org.
