# Supplementary material for: Why are some countries rich and others poor? development and validation of the attributions for Cross-Country Inequality Scale (ACIS)
Source: PLoS One. 2024 Feb 27;19(2):e0298222. doi: 10.1371/journal.pone.0298222 (PMC10898736; doi:10.1371/journal.pone.0298222)
Supplement: S1 Table — (DOCX) [file pone.0298222.s002.docx]

**Why are poor countries poorer than rich countries?**

**Development and validation of the Cross-Country Inequality Attributions Scale (C-CIAS)**

**Supplementary Materials**

**Table S1.** Descriptive statistics of the samples and measures by country (Study 1).

|  | **Italy (N = 246)** | **South Africa (N = 228)** | **UK (N = 248)** |
| --- | --- | --- | --- |
| **Age** | 27.35 (8.03) | 27.33 (7.73) | 40.57 (15.22) |
| **Education** | 17.25 (2.75) | 15.91 (3.14) | 16.49 (9.63) |
| **Gender – *n* (%)** |  |  |  |
| *Female* | 122 (50%) | 112 (49%) | 122 (49%) |
| *Male* | 117 (48%) | 115 (50%) | 124 (50%) |
| *Non-binary* | 7 (2.8%) | 1 (0.4%) | 2 (0.8%) |
| **Work status – *n* (%)** |  |  |  |
| *Employed full-time (30+ hours weekly)* | 54 (22%) | 89 (39%) | 113 (46%) |
| *Employed part-time (15-29 hours weekly)* | 41 (17%) | 21 (9.2%) | 37 (15%) |
| *Employed < 15 hours weekly* | 15 (6.1%) | 4 (1.8%) | 14 (5.6%) |
| *Apprentice or trainee* | 4 (1.6%) | 7 (3.1%) | 0 (0%) |
| *Unemployed and looking for a job* | 22 (8.9%) | 39 (17%) | 10 (4.0%) |
| *Unable to join the work force* | 0 (0%) | 1 (0.4%) | 8 (3.2%) |
| *Student* | 109 (44%) | 63 (28%) | 28 (11%) |
| *Housekeeping* | 1 (0.4%) | 3 (1.3%) | 13 (5.2%) |
| *Retired* | 0 (0%) | 1 (0.4%) | 25 (10%) |
| **Inequality perception** | 4.51 (0.73) | 4.55 (0.82) | 4.27 (0.92) |
| **Redistribution** | 3.51 (1.22) | 3.48 (1.30) | 3.23 (1.25) |
| **Migration** | 4.01 (1.01) | 4.39 (0.91) | 3.79 (1.12) |
| **Unfairness** | 4.27 (0.89) | 4.04 (1.01) | 4.08 (0.95) |
| **Moralization** | 4.26 (0.83) | 4.10 (0.94) | 3.95 (1.00) |
| **Moral outrage** | 3.78 (0.96) | 3.76 (1.06) | 3.27 (1.03) |
| **Meritocracy** | 2.90 (0.77) | 3.53 (0.80) | 2.95 (0.92) |
| **Social dominance** | 1.69 (0.68) | 1.59 (0.64) | 1.76 (0.76) |
| **Economic system justification** | 2.22 (0.64) | 2.41 (0.60) | 2.39 (0.75) |
| **Position own country – *n* (%)** |  |  |  |
| *Poorest 20%* | 1 (0.4%) | 2 (0.9%) | 1 (0.4%) |
| *Second poorest 20%* | 16 (6.5%) | 8 (3.5%) | 1 (0.4%) |
| *Middle 20%* | 95 (39%) | 116 (51%) | 35 (14%) |
| *Second richest 20%* | 78 (32%) | 86 (38%) | 77 (31%) |
| *Richest 20%* | 56 (23%) | 16 (7.0%) | 134 (54%) |
| **Political orientation** | 3.79 (1.83) | 5.39 (2.21) | 5.21 (2.16) |
| **National identity** | 2.74 (0.83) | 2.72 ( 0.97) | 2.93 (0.98) |
| **Subjective SES** | 5.48 (1.43) | 4.61 (1.51) | 5.09 (1.79) |
| **Life satisfaction** | 4.31 (1.41) | 4.06 (1.52) | 4.46 (1.40) |
| **Horizontal trust** | 2.13 (0.80) | 1.53 (0.73) | 2.26 (0.75) |

*Notes.* Means and standard deviations are reported for all the variables, but for categorical variables.
