# Supplementary material for: Why are some countries rich and others poor? development and validation of the attributions for Cross-Country Inequality Scale (ACIS)
Source: PLoS One. 2024 Feb 27;19(2):e0298222. doi: 10.1371/journal.pone.0298222 (PMC10898736; doi:10.1371/journal.pone.0298222)
Supplement: S2 Table — (DOCX) [file pone.0298222.s003.docx]

**Table S2**. Correlations for the Italian sample (Study 1; n = 246).

| **Variable** | | **α** | **1** | **2** | **3** | **4** | **5** | **6** | **7** | **8** | **9** | **10** | **11** | **12** | **13** | **14** | **15** | **16** | **17** | **18** | **19** | **20** |
| --- | --- | --- | --- | --- | --- | --- | --- | --- | --- | --- | --- | --- | --- | --- | --- | --- | --- | --- | --- | --- | --- | --- |
| **1** | **Rich countries** | .88 |  |  |  |  |  |  |  |  |  |  |  |  |  |  |  |  |  |  |  |  |
| **2** | **Poor countries** | .86 | -.25** |  |  |  |  |  |  |  |  |  |  |  |  |  |  |  |  |  |  |  |
| **3** | **Fate** | .72 | -.13* | .51** |  |  |  |  |  |  |  |  |  |  |  |  |  |  |  |  |  |  |
| **4** | **Inequality perception** |  | .40** | -.37** | -.27** |  |  |  |  |  |  |  |  |  |  |  |  |  |  |  |  |  |
| **5** | **Redistribution** |  | .35** | -.27** | -.08 | .28** |  |  |  |  |  |  |  |  |  |  |  |  |  |  |  |  |
| **6** | **Migration** |  | .27** | -.27** | -.00 | .20** | .30** |  |  |  |  |  |  |  |  |  |  |  |  |  |  |  |
| **7** | **Unfairness** |  | .38** | .30** | -.24** | .49** | -.09 | -.00 |  |  |  |  |  |  |  |  |  |  |  |  |  |  |
| **8** | **Moralization** |  | .47** | -.32** | -.19** | .49** | .38** | .38** | .34** |  |  |  |  |  |  |  |  |  |  |  |  |  |
| **9** | **Moral outrage** |  | .39** | -.32** | -.15* | .44** | .49** | .38** | .28** | .60** |  |  |  |  |  |  |  |  |  |  |  |  |
| **10** | **Meritocracy** | .84 | -.24** | .45** | .30** | -.28** | -.18** | -.18** | -.31** | -.31** | -.34** |  |  |  |  |  |  |  |  |  |  |  |
| **11** | **SDO** | .69 | -.44** | .48** | .41** | -.55** | -.33** | -.29** | -.37** | -.52** | -.52** | .33** |  |  |  |  |  |  |  |  |  |  |
| **12** | **ESJ** | .80 | -.53** | .49** | .35** | -.39** | -.36** | -.26** | -.41** | -.56** | -.50** | .54** | .53** |  |  |  |  |  |  |  |  |  |
| **13** | **Country SES** |  | -.10 | -.12 | -.05 | .14* | -.10 | -.11 | -.21** | -.02 | -.08 | -.10 | .02 | -.07 |  |  |  |  |  |  |  |  |
| **14** | **Political orientation** |  | -.27** | .43** | .16* | -.35** | -.26** | -.23** | -.27** | -.32** | -.38** | .41** | .38** | .40** | -.15* |  |  |  |  |  |  |  |
| **15** | **National identity** | .69 | -.19** | .21** | .22** | -.08 | -.19** | -.21** | -.07 | -.15* | -.15* | .18** | .15* | .24** | .36** | .07 |  |  |  |  |  |  |
| **16** | **SSES** |  | -.16* | -.04 | -.04 | -.10 | -.03 | .11 | -.02 | .00 | -.10 | .01 | .09 | .09 | .23** | .02 | .17** |  |  |  |  |  |
| **17** | **Life satisfaction** |  | -.05 | -.01 | -.04 | .02 | -.01 | .01 | .03 | -.03 | -.10 | .02 | .05 | .08 | .17** | -.01 | .24** | .46** |  |  |  |  |
| **18** | **Horizontal trust** |  | -.09 | -.09 | -.03 | .02 | .11 | .03 | .00 | .04 | .06 | -.11 | -.05 | -.05 | .20** | -.07 | .28** | .11 | .20** |  |  |  |
| **19** | **Age** |  | -.01 | .11 | -.10 | .09 | .01 | -.21** | .10 | .08 | .01 | -.00 | .01 | -.16* | .01 | .20** | -.01 | -.10 | -.05 | .09 |  |  |
| **20** | **Gender** |  | .23** | -.27** | -.19** | .16* | .20** | .27** | .08 | .23** | .27** | -.18** | -.25** | -.20** | -.18** | -.25** | -.12 | -.05 | -.05 | -.08 | -.12 |  |
| **21** | **Education** |  | -.05 | .06 | .04 | -.02 | -.10 | -.04 | .04 | -.02 | -.15* | -.00 | .01 | .06 | .09 | .01 | .02 | .03 | .13* | .01 | .19** | -.05 |

*Note.* SDO = Social Dominance Orientation; ESJ = Economic System Justification; SSES = Subjective Socioeconomic Status. ** *p* < .001, * *p* < .05
