# Supplementary material for: Why are some countries rich and others poor? development and validation of the attributions for Cross-Country Inequality Scale (ACIS)
Source: PLoS One. 2024 Feb 27;19(2):e0298222. doi: 10.1371/journal.pone.0298222 (PMC10898736; doi:10.1371/journal.pone.0298222)
Supplement: S3 Table — (DOCX) [file pone.0298222.s004.docx]

**Table S3**. Correlations for the South African sample (Study 1; n = 228).

| **Variable** | | **α** | **1** | **2** | **3** | **4** | **5** | **6** | **7** | **8** | **9** | **10** | **11** | **12** | **13** | **14** | **15** | **16** | **17** | **18** | **19** | **20** |
| --- | --- | --- | --- | --- | --- | --- | --- | --- | --- | --- | --- | --- | --- | --- | --- | --- | --- | --- | --- | --- | --- | --- |
| **1** | **Rich countries** | .86 |  |  |  |  |  |  |  |  |  |  |  |  |  |  |  |  |  |  |  |  |
| **2** | **Poor countries** | .84 | -.16* |  |  |  |  |  |  |  |  |  |  |  |  |  |  |  |  |  |  |  |
| **3** | **Fate** | .74 | -.15* | .50** |  |  |  |  |  |  |  |  |  |  |  |  |  |  |  |  |  |  |
| **4** | **Inequality perception** |  | .38** | -.17* | -.15* |  |  |  |  |  |  |  |  |  |  |  |  |  |  |  |  |  |
| **5** | **Redistribution** |  | .28** | -.08 | .08 | .21** |  |  |  |  |  |  |  |  |  |  |  |  |  |  |  |  |
| **6** | **Migration** |  | .39** | -.13* | -.20** | .22** | .22** |  |  |  |  |  |  |  |  |  |  |  |  |  |  |  |
| **7** | **Unfairness** |  | .22** | -.15* | -.19** | .17** | .19** | .07 |  |  |  |  |  |  |  |  |  |  |  |  |  |  |
| **8** | **Moralization** |  | .35** | -.16* | -.19** | .30** | .33** | .23** | .33** |  |  |  |  |  |  |  |  |  |  |  |  |  |
| **9** | **Moral outrage** |  | .39** | -.07 | -.03 | .16* | .43** | .25** | .25** | .48** |  |  |  |  |  |  |  |  |  |  |  |  |
| **10** | **Meritocracy** | .79 | .05 | .33** | .15* | .00 | -.01 | .07 | -.15* | -.08 | -.10 |  |  |  |  |  |  |  |  |  |  |  |
| **11** | **SDO** | .48 | -.34** | .23** | .17* | -.17* | -.14* | -.19** | -.17* | -.25** | -.17** | .08 |  |  |  |  |  |  |  |  |  |  |
| **12** | **ESJ** | .67 | -.38** | .35** | .30** | -.19** | -.23** | -.23** | -.24** | -.36** | -.36** | .28** | .42** |  |  |  |  |  |  |  |  |  |
| **13** | **Country SES** |  | -.08 | .10 | .01 | -.07 | -.11 | .07 | -.10 | -.10 | .00 | .12 | .02 | .04 |  |  |  |  |  |  |  |  |
| **14** | **Political orientation** |  | -.05 | .15* | .11 | .03 | -.18** | .00 | -.05 | -.07 | -.17** | .19** | .15* | .18** | .09 |  |  |  |  |  |  |  |
| **15** | **National identity** | .69 | .09 | .00 | -.03 | .06 | .06 | .01 | -.06 | .06 | .11 | .06 | -.07 | -.04 | .31** | .00 |  |  |  |  |  |  |
| **16** | **Subjective SES** |  | .06 | -.04 | -.01 | .04 | .01 | .03 | -.01 | -.11 | .04 | -.01 | -.10 | -.02 | .17* | .07 | .24** |  |  |  |  |  |
| **17** | **Life satisfaction** |  | -.01 | .05 | -.02 | -.04 | .05 | -.07 | -.03 | -.13 | -.05 | .09 | .07 | .18** | .15* | .10 | .26** | .41** |  |  |  |  |
| **18** | **Horizontal trust** |  | -.18** | -.04 | -.07 | -.08 | .03 | -.04 | .05 | -.08 | -.13 | -.09 | -.03 | .07 | .02 | -.07 | .06 | .07 | .18** |  |  |  |
| **19** | **Age** |  | .03 | -.03 | -.09 | .01 | .04 | .02 | .01 | .02 | -.06 | -.01 | -.07 | -.05 | -.06 | .06 | .09 | -.12 | -.07 | .14* |  |  |
| **20** | **Gender** |  | .03 | -.02 | -.05 | -.04 | -.03 | -.03 | .00 | -.09 | .04 | -.20** | -.09 | -.11 | -.18** | -.16* | -.17* | .05 | -.10 | -.05 | -.00 |  |
| **21** | **Education** |  | .12 | -.20** | -.15* | .08 | -.02 | .11 | .13 | .06 | .11 | -.13* | -.00 | -.12 | -.09 | .02 | -.05 | .05 | -.03 | -.10 | -.04 | .05 |

*Note.* SDO = Social Dominance Orientation; ESJ = Economic System Justification; SSES = Subjective Socioeconomic Status. ** *p* < .001, * *p* < .05.
