# Supplementary material for: Why are some countries rich and others poor? development and validation of the attributions for Cross-Country Inequality Scale (ACIS)
Source: PLoS One. 2024 Feb 27;19(2):e0298222. doi: 10.1371/journal.pone.0298222 (PMC10898736; doi:10.1371/journal.pone.0298222)
Supplement: S4 Table — (DOCX) [file pone.0298222.s005.docx]

**Table S4.** Correlations for the British sample (Study 1; n = 248).

| **Variable** | | **α** | **1** | **2** | **3** | **4** | **5** | **6** | **7** | **8** | **9** | **10** | **11** | **12** | **13** | **14** | **15** | **16** | **17** | **18** | **19** | **20** |
| --- | --- | --- | --- | --- | --- | --- | --- | --- | --- | --- | --- | --- | --- | --- | --- | --- | --- | --- | --- | --- | --- | --- |
| **1** | **Rich countries** | .91 |  |  |  |  |  |  |  |  |  |  |  |  |  |  |  |  |  |  |  |  |
| **2** | **Poor countries** | .83 | -.29** |  |  |  |  |  |  |  |  |  |  |  |  |  |  |  |  |  |  |  |
| **3** | **Fate** | .72 | .03 | .38** |  |  |  |  |  |  |  |  |  |  |  |  |  |  |  |  |  |  |
| **4** | **Inequality perception** |  | .54** | -.34** | -.01 |  |  |  |  |  |  |  |  |  |  |  |  |  |  |  |  |  |
| **5** | **Redistribution** |  | .44** | -.20** | .07 | .47** |  |  |  |  |  |  |  |  |  |  |  |  |  |  |  |  |
| **6** | **Migration** |  | .36** | -.18** | -.04 | .24** | .43** |  |  |  |  |  |  |  |  |  |  |  |  |  |  |  |
| **7** | **Unfairness** |  | .49** | -.35** | -.01 | .51** | .33** | .14* |  |  |  |  |  |  |  |  |  |  |  |  |  |  |
| **8** | **Moralization** |  | .47** | -.36** | -.07 | .50** | .53** | .36** | .43** |  |  |  |  |  |  |  |  |  |  |  |  |  |
| **9** | **Moral outrage** |  | .50** | -.34** | -.05 | .50** | .57** | .42** | .45** | .60** |  |  |  |  |  |  |  |  |  |  |  |  |
| **10** | **Meritocracy** | .88 | -.29** | .53** | .22** | -.26** | -.25** | -.10 | -.33** | -.30** | -.41** |  |  |  |  |  |  |  |  |  |  |  |
| **11** | **SDO** | .71 | -.45** | .38** | .17** | -.37** | -.42** | -.23** | -.30** | -.46** | -.44** | .39** |  |  |  |  |  |  |  |  |  |  |
| **12** | **ESJ** | .84 | -.50** | .52** | .26** | -.37** | -.40** | -.26** | -.43** | -.44** | -.54** | .66** | .62** |  |  |  |  |  |  |  |  |  |
| **13** | **Country SES** |  | -.08 | -.16* | -.18** | .03 | -.05 | -.11 | .10 | .06 | -.03 | -.17** | .02 | -.13* |  |  |  |  |  |  |  |  |
| **14** | **Political orientation** |  | -.47** | .38** | .20** | -.26** | -.36** | -.28** | -.33** | -.39** | -.45** | .49** | .44** | .61** | -.18** |  |  |  |  |  |  |  |
| **15** | **National identity** | .76 | -.27** | .30** | .22** | -.11 | -.20** | -.27** | -.22** | -.24** | -.24** | .42** | .18** | .40** | -.12 | .44** |  |  |  |  |  |  |
| **16** | **Subjective SES** |  | -.31** | -.01 | -.06 | -.17** | -.06 | -.08 | -.10 | -.08 | -.15* | .12* | .24** | .23** | .21** | .06 | .07 |  |  |  |  |  |
| **17** | **Life satisfaction** |  | -.17** | -.00 | .06 | -.14* | .07 | -.04 | -.12 | -.03 | -.11 | .11 | .05 | .17** | .10 | .07 | .25** | .51** |  |  |  |  |
| **18** | **Horizontal trust** |  | .03 | -.19** | -.03 | .05 | .16* | .01 | .08 | .10 | .11 | -.19** | -.08 | -.17** | .13* | -.17** | .05 | .22** | .20** |  |  |  |
| **19** | **Age** |  | -.07 | .09 | .05 | -.02 | -.04 | -.23** | -.01 | -.13* | -.20** | .03 | .12 | .11 | -.10 | .27** | -.07 | -.01 | .05 | .05 |  |  |
| **20** | **Gender** |  | .10 | -.13* | .01 | .06 | .13* | .16* | .04 | .21** | .21** | -.09 | -.19** | -.11 | -.23** | -.07 | -.10 | -.04 | .07 | -.06 | -.02 |  |
| **21** | **Education** |  | .09 | -.02 | .06 | -.20** | -.12 | .08 | .01 | .10 | .04 | -.04 | .10 | -.03 | .03 | -.12 | -.02 | .06 | .09 | -.01 | -.10 | .10 |

*Note.* SDO = Social Dominance Orientation; ESJ = Economic System Justification; SSES = Subjective Socioeconomic Status. ** *p* < .001, * *p* < .05
