# Supplementary material for: Why are some countries rich and others poor? development and validation of the attributions for Cross-Country Inequality Scale (ACIS)
Source: PLoS One. 2024 Feb 27;19(2):e0298222. doi: 10.1371/journal.pone.0298222 (PMC10898736; doi:10.1371/journal.pone.0298222)
Supplement: S5 Table — (DOCX) [file pone.0298222.s006.docx]

**Table S5.** Regression results of causal attributions on inequality appraisals, Study 1 (N = 722).

|  | **Inequality Perception** | | | | | | **Redistribution** | | | | | |
| --- | --- | --- | --- | --- | --- | --- | --- | --- | --- | --- | --- | --- |
|  | **Model 1 (R^2^ adjusted = .226)** | | | **Model 2 (R^2^ adjusted = .221)** | | | **Model 1 (R^2^ adjusted = .145)** | | | **Model 2 (R^2^ adjusted = .141)** | | |
| **Predictors** | **Beta** | **95% CI** | ***p*** | **Beta** | **95% CI** | ***p*** | **Beta** | **95% CI** | ***p*** | **Beta** | **95% CI** | ***p*** |
| (Intercept) | 2.73 | 2.29 – 3.18 | **<.001** | 0.08 | -0.06 – 0.22 | **<.001** | -0.01 | -0.14 – 0.12 | **.018** | 0.04 | -0.11 – 0.19 | **.027** |
| Rich countries | 0.51 | 0.42 – 0.59 | **<.001** | 0.42 | 0.35 – 0.49 | **<.001** | 0.35 | 0.27 – 0.42 | **<.001** | 0.34 | 0.27 – 0.41 | **<.001** |
| Poor countries | -0.17 | -0.24 – -0.10 | **<.001** | -0.19 | -0.27 – -0.11 | **<.001** | -0.17 | -0.25 – -0.08 | **<.001** | -0.16 | -0.24 – -0.08 | **<.001** |
| Fate | -0.02 | -0.09 – 0.06 | .677 | -0.01 | -0.09 – 0.06 | .728 | 0.12 | 0.05 – 0.20 | **.002** | 0.13 | 0.05 – 0.20 | **.001** |
| Country [ita] | 0.01 | -0.14 – 0.15 | .935 | 0.01 | -0.16 – 0.18 | .891 | 0.11 | -0.07 – 0.29 | .228 | 0.11 | -0.07 – 0.29 | .247 |
| Country [uk] | -0.17 | -0.31 – -0.02 | **.023** | -0.25 | -0.44 – -0.06 | **.011** | -0.09 | -0.27 – 0.09 | .343 | -0.11 | -0.31 – 0.09 | .279 |
| Gender [male] |  |  |  | 0.01 | -0.12 – 0.14 | .896 |  |  |  | -0.08 | -0.22 – 0.06 | .248 |
| Gender [non-binary] |  |  |  | -0.38 | -0.93 – 0.17 | .171 |  |  |  | 0.30 | -0.28 – 0.89 | .305 |
| Age |  |  |  | 0.04 | -0.03 – 0.12 | .254 |  |  |  | 0.02 | -0.06 – 0.10 | .571 |
|  | *F*(3,713) = 1.12, *p* = .338 | | | | | | *F*(3,713) = 2.59, *p* = .338 | | | | | |
|  | **Migration** | | | | | | **Unfairness** | | | | | |
|  | **Model 1 (R^2^ adjusted = .241)** | | | **(R^2^ adjusted = .223)** | | | **Model 1 (R^2^ adjusted = .193)** | | | **Model 2 (R^2^ adjusted = .194)** | | |
|  | **Beta** | **95% CI** | ***p*** | **Beta** | **95% CI** | ***p*** | **Beta** | **95% CI** | ***p*** | **Beta** | **95% CI** | ***p*** |
| (Intercept) | 0.26 | 0.13 – 0.39 | **<.001** | 0.12 | -0.02 – 0.27 | **<.001** | -0.14 | -0.27 – -0.01 | **<.001** | -0.13 | -0.28 – 0.02 | **<.001** |
| Rich countries | 0.31 | 0.24 – 0.38 | **<.001** | 0.30 | 0.23 – 0.37 | **<.001** | 0.34 | 0.27 – 0.41 | **<.001** | 0.34 | 0.27 – 0.42 | **<.001** |
| Poor countries | -0.12 | -0.20 – -0.04 | **.005** | -0.09 | -0.18 – -0.01 | **.023** | -0.16 | -0.24 – -0.08 | **<.001** | -0.17 | -0.25 – -0.08 | **<.001** |
| Fate | -0.01 | -0.08 – 0.07 | .870 | -0.01 | -0.09 – 0.06 | .703 | -0.05 | -0.13 – 0.03 | .193 | -0.05 | -0.12 – 0.03 | .215 |
| Country [ita] | -0.30 | -0.48 – -0.12 | **.001** | -0.31 | -0.48 – -0.13 | **.001** | 0.26 | 0.08 – 0.44 | **.004** | 0.26 | 0.08 – 0.44 | **.004** |
| Country [uk] | -0.46 | -0.64 – -0.28 | **<.001** | -0.28 | -0.47 – -0.08 | **.006** | 0.14 | -0.04 – 0.32 | .135 | 0.09 | -0.11 – 0.29 | .374 |
| Gender [male] |  |  |  | 0.13 | -0.00 – 0.27 | .053 |  |  |  | 0.03 | -0.11 – 0.17 | .666 |
| Gender [non-binary] |  |  |  | 0.66 | 0.09 – 1.23 | **.024** |  |  |  | -0.27 | -0.85 – 0.30 | .351 |
| Age |  |  |  | -0.17 | -0.24 – -0.09 | **<.001** |  |  |  | 0.04 | -0.03 – 0.12 | .275 |
|  | *F*(3,713) = 9.11, *p* < .001 | | | | | | *F*(3,713) = 0.82, *p* = .484 | | | | | |
|  | **Moralization** | | | | | | **Moral Outrage** | | | | | |
|  | **Model 1 (R^2^ adjusted = .234)** | | | **Model 2 (R^2^ adjusted = .235)** | | | **Model 1 (R^2^ adjusted = .291)** | | | **Model 2 (R^2^ adjusted = .284)** | | |
|  | **Beta** | **95% CI** | ***p*** | **Beta** | **95% CI** | ***p*** | **Beta** | **95% CI** | ***p*** | **Beta** | **95% CI** | ***p*** |
| (Intercept) | -0.06 | -0.18 – 0.06 | **<.001** | -0.04 | -0.18 – 0.10 | **<.001** | 0.08 | -0.05 – 0.20 | **<.001** | 0.13 | -0.01 – 0.27 | **<.001** |
| Rich countries | 0.40 | 0.34 – 0.47 | **<.001** | 0.40 | 0.33 – 0.47 | **<.001** | 0.40 | 0.33 – 0.47 | **<.001** | 0.39 | 0.32 – 0.46 | **<.001** |
| Poor countries | -0.17 | -0.25 – -0.09 | **<.001** | -0.17 | -0.25 – -0.09 | **<.001** | -0.15 | -0.23 – -0.07 | **<.001** | -0.13 | -0.21 – -0.05 | **.001** |
| Fate | -0.04 | -0.11 – 0.03 | .288 | -0.04 | -0.11 – 0.03 | .277 | 0.03 | -0.05 – 0.10 | .467 | 0.02 | -0.05 – 0.10 | .519 |
| Country [ita] | 0.21 | 0.04 – 0.39 | **.015** | 0.21 | 0.04 – 0.38 | **.017** | 0.11 | -0.07 – 0.28 | .226 | 0.11 | -0.06 – 0.28 | .221 |
| Country [uk] | -0.04 | -0.22 – 0.13 | .653 | -0.01 | -0.20 – 0.18 | .901 | -0.32 | -0.50 – -0.15 | **<.001** | -0.23 | -0.42 – -0.03 | **.020** |
| Gender [male] |  |  |  | -0.06 | -0.19 – 0.07 | .350 |  |  |  | -0.19 | -0.32 – -0.06 | **.004** |
| Gender [non-binary] |  |  |  | 0.36 | -0.19 – 0.92 | .200 |  |  |  | 0.36 | -0.19 – 0.91 | .196 |
| Age |  |  |  | -0.02 | -0.10 – 0.05 | .523 |  |  |  | -0.09 | -0.16 – -0.02 | **.017** |
|  | *F*(3,713) = 1.11, *p* = .342 | | | | | | *F*(3,713) = 5.80, *p* < .001 | | | | | |

*Notes.* Country reference level is South Africa. Gender reference level is female
