# Supplementary material for: Why are some countries rich and others poor? development and validation of the attributions for Cross-Country Inequality Scale (ACIS)
Source: PLoS One. 2024 Feb 27;19(2):e0298222. doi: 10.1371/journal.pone.0298222 (PMC10898736; doi:10.1371/journal.pone.0298222)
Supplement: S6 Table — (DOCX) [file pone.0298222.s007.docx]

**Table S6.** Descriptive statistics of the samples and measures by country (Study 2).

|  | **Italy**  **(N = 239)** | **South Africa**  **(N = 248)** | **UK**  **(N = 249)** |
| --- | --- | --- | --- |
| **Age** | 27.14 (7.21) | 28.52 (8.10) | 42.20 (13.11) |
| **Education** | 16.94 (2.64) | 15.71 (3.14) | 15.96 (3.30) |
| **Gender – *n* (%)** |  |  |  |
| *Female* | 114 (48%) | 124 (50%) | 123 (49%) |
| *Male* | 120 (50%) | 123 (50%) | 125 (50%) |
| *Non-binary* | 5 (2.1%) | 1 (0.4%) | 1 (0.4%) |
| **Work status – *n* (%)** |  |  |  |
| *Employed full-time (30+ hours weekly)* | 62 (27%) | 109 (45%) | 122 (49%) |
| *Employed part-time (15-29 hours weekly)* | 17 (7.3%) | 22 (9.1%) | 41 (16%) |
| *Employed < 15 hours weekly* | 8 (3.4%) | 7 (2.9%) | 13 (5.2%) |
| *Apprentice or trainee* | 4 (1.7%) | 8 (3.3%) | 0 (0%) |
| *Unemployed and looking for a job* | 26 (11%) | 27 (11%) | 11 (4.4%) |
| *Unable to join the work force* | 0 (0%) | 1 (0.4%) | 10 (4.0%) |
| *Student* | 108 (46%) | 62 (26%) | 9 (3.6%) |
| *Housekeeping* | 1 (0.4%) | 4 (1.6%) | 22 (8.8%) |
| *Retired* | 7 (3.0%) | 3 (1.2%) | 21 (8.4%) |
| **Rich countries** | 4.21 (0.60) | 4.40 (0.69) | 3.96 (0.75) |
| **Poor countries** | 2.00 (0.74) | 2.90 (0.92) | 2.08 (0.80) |
| **Fate** | 1.80 (0.77) | 1.98 (0.94) | 2.15 (0.81) |
| **Inequality perception** | 4.59 (0.62) | 4.69 (0.67) | 4.43 (0.81) |
| **Redistribution** | 3.33 (1.19) | 3.52 (1.34) | 3.16 (1.27) |
| **Migration** | 4.08 (0.90) | 4.50 (0.84) | 3.83 (1.01) |
| **Unfairness** | 4.19 (0.83) | 4.02 (1.18) | 4.03 (0.89) |
| **Moralization** | 4.23 (0.78) | 4.10 (0.96) | 3.89 (0.90) |
| **Moral outrage** | 3.69 (0.85) | 3.73 (1.09) | 3.18 (1.01) |
| **Position own country – *n* (%)** |  |  |  |
| *Poorest 20%* | 9 (3.8%) | 25 (10%) | 10 (4.0%) |
| *Second poorest 20%* | 11 (4.6%) | 73 (29%) | 5 (2.0%) |
| *Middle 20%* | 69 (29%) | 127 (51%) | 37 (15%) |
| *Second richest 20%* | 99 (41%) | 16 (6.5%) | 83 (33%) |
| *Richest 20%* | 51 (21%) | 7 (2.8%) | 114 (46%) |
| **Meritocracy** | 2.91 (0.70) | 3.51 (0.80) | 3.02 (0.82) |
| **Social dominance** | 1.68 (0.64) | 1.59 (0.65) | 1.77 (0.73) |
| **Economic system justification** | 2.17 (0.60) | 2.40 (0.58) | 2.36 (0.68) |
| **Zero sum beliefs** | 3.59 (0.87) | 3.91 (1.08) | 3.39 (1.02) |
| **Perceived need of international institutions** | 4.22 (0.91) | 4.25 (1.08) | 4.02 (0.99) |
| **Trust in international institutions** | 2.76 (1.16) | 3.20 (0.91) | 3.17 (0.97) |
| **Political orientation** | 3.98 (1.93) | 5.53 (2.53) | 5.04 (2.05) |
| **Subjective SES** | 5.77 (1.41) | 4.92 (1.51) | 5.33 (1.59) |
| **Self-identification with own country** | 3.97 (1.51) | 4.17 (1.91) | 4.05 (1.57) |
| **Self-identification with world population** | 4.09 (1.45) | 2.94 (1.58) | 3.26 (1.36) |
| **Horizontal trust** | 2.63 (0.90) | 2.01 (1.05) | 2.79 (0.98) |

*Notes.* Means and standard deviations are reported for all the variables, but for categorical variables.
