# Supplementary material for: Why are some countries rich and others poor? development and validation of the attributions for Cross-Country Inequality Scale (ACIS)
Source: PLoS One. 2024 Feb 27;19(2):e0298222. doi: 10.1371/journal.pone.0298222 (PMC10898736; doi:10.1371/journal.pone.0298222)
Supplement: S7 Table — (DOCX) [file pone.0298222.s008.docx]

**Table S7.** Correlations for the Italian sample (Study 2; n = 239).

| **Variable** | | **α** | **1** | **2** | **3** | **4** | **5** | **6** | **7** | **8** | **9** | **10** | **11** | **12** | **13** | **14** | **15** | **16** | **17** | **18** | **19** | **20** | **21** | **22** | **23** | **24** |
| --- | --- | --- | --- | --- | --- | --- | --- | --- | --- | --- | --- | --- | --- | --- | --- | --- | --- | --- | --- | --- | --- | --- | --- | --- | --- | --- |
| **1** | **Rich countries** | .88 |  |  |  |  |  |  |  |  |  |  |  |  |  |  |  |  |  |  |  |  |  |  |  |  |
| **2** | **Poor countries** | .83 | -.47** |  |  |  |  |  |  |  |  |  |  |  |  |  |  |  |  |  |  |  |  |  |  |  |
| **3** | **Fate** | .69 | -.26** | .34** |  |  |  |  |  |  |  |  |  |  |  |  |  |  |  |  |  |  |  |  |  |  |
| **4** | **Inequality perception** |  | .38** | -.25** | -.24** |  |  |  |  |  |  |  |  |  |  |  |  |  |  |  |  |  |  |  |  |  |
| **5** | **Redistribution** |  | .25** | -.17** | -.11 | .23** |  |  |  |  |  |  |  |  |  |  |  |  |  |  |  |  |  |  |  |  |
| **6** | **Migration** |  | .34** | -.29** | -.11 | .32** | .33** |  |  |  |  |  |  |  |  |  |  |  |  |  |  |  |  |  |  |  |
| **7** | **Unfairness** |  | .36** | -.18** | -.16* | .38** | .10 | .18** |  |  |  |  |  |  |  |  |  |  |  |  |  |  |  |  |  |  |
| **8** | **Morality** |  | .38** | -.27** | -.23** | .42** | .36** | .29** | .33** |  |  |  |  |  |  |  |  |  |  |  |  |  |  |  |  |  |
| **9** | **Moral outrage** |  | .35** | -.22** | -.30** | .37** | .30** | .33** | .26** | .46** |  |  |  |  |  |  |  |  |  |  |  |  |  |  |  |  |
| **10** | **Country SES** |  | -.00 | -.04 | -.04 | .12 | -.04 | -.03 | .04 | .03 | -.02 |  |  |  |  |  |  |  |  |  |  |  |  |  |  |  |
| **11** | **Need for**  **institutions** |  | .18** | -.23** | -.15* | .30** | .14* | .30** | .11 | .20** | .23** | .13* |  |  |  |  |  |  |  |  |  |  |  |  |  |  |
| **12** | **Trust in**  **institutions** |  | -.16* | -.09 | -.01 | .07 | .14 | .20** | -.14 | -.03 | .02 | .13 | .44** |  |  |  |  |  |  |  |  |  |  |  |  |  |
| **13** | **Horizontal trust** |  | .03 | -.10 | .02 | .13* | .14* | .12 | -.07 | .08 | .02 | .11 | .21** | .28** |  |  |  |  |  |  |  |  |  |  |  |  |
| **14** | **Zero sum beliefs** | .80 | .51** | -.15* | -.15* | .18** | .14* | .24** | .16* | .30** | .25** | -.04 | .06 | -.07 | -.03 |  |  |  |  |  |  |  |  |  |  |  |
| **15** | **Meritocracy** | .80 | -.26** | .41** | .26** | -.15* | -.09 | -.13 | -.17** | -.19** | -.30** | -.03 | -.02 | .24** | .06 | -.06 |  |  |  |  |  |  |  |  |  |  |
| **16** | **SDO** | .78 | -.45** | .42** | .32** | -.34** | -.24** | -.38** | -.25** | -.40** | -.37** | -.02 | -.30** | -.08 | -.03 | -.23** | .28** |  |  |  |  |  |  |  |  |  |
| **17** | **ESJ** | .79 | -.52** | .55** | .45** | -.42** | -.20** | -.27** | -.34** | -.53** | -.43** | -.01 | -.22** | .11 | -.05 | -.19** | .51** | .57** |  |  |  |  |  |  |  |  |
| **18** | **Country mobility** | .45 | -.25** | .18** | .06 | -.14* | -.09 | -.14* | -.13* | -.15* | -.14* | -.02 | -.19** | .05 | .06 | -.15* | .13* | .16* | .17** |  |  |  |  |  |  |  |
| **19** | **Self-identification country** |  | -.02 | .09 | -.06 | .09 | .08 | -.08 | -.07 | .02 | .01 | .16* | .10 | .11 | .14* | -.02 | .11 | -.09 | -.00 | .05 |  |  |  |  |  |  |
| **20** | **Self-identification world** |  | .10 | -.20** | -.12 | .12 | .16* | .05 | .01 | .08 | .07 | .19** | .25** | .28** | .24** | .07 | .00 | -.20** | -.10 | .01 | .20** |  |  |  |  |  |
| **21** | **SSES** |  | -.07 | -.01 | .05 | .00 | -.05 | -.12 | -.14* | -.08 | -.12 | .26** | .17** | .22** | .16* | -.11 | .11 | .04 | .09 | .03 | .32** | .14* |  |  |  |  |
| **22** | **Political orientation** |  | -.26** | .39** | .27** | -.25** | -.20** | -.23** | -.20** | -.24** | -.28** | .06 | -.26** | -.17* | -.05 | -.12 | .29** | .45** | .43** | .15* | .02 | -.15* | .03 |  |  |  |
| **23** | **Age** |  | -.09 | .20** | .00 | -.05 | -.01 | -.22** | -.11 | .06 | -.09 | -.04 | -.13* | -.21** | .06 | -.07 | -.00 | .17* | .08 | .00 | .07 | .07 | .07 | .16* |  |  |
| **24** | **Gender** |  | .20** | -.27** | -.19** | .09 | .18** | .18** | .07 | .22** | .26** | -.11 | .10 | .07 | -.16* | .26** | -.12 | -.32** | -.18** | -.10 | -.01 | .06 | -.09 | -.22** | -.16* |  |
| **25** | **Education** |  | .01 | -.04 | .01 | .10 | .01 | .05 | .04 | .01 | -.02 | .10 | .14* | .07 | .07 | -.01 | .03 | -.02 | .03 | .17** | .13* | .09 | .13 | -.10 | .09 | .13* |

*Note.* SDO = Social Dominance Orientation; ESJ = Economic System Justification; SSES = Subjective Socioeconomic Status. ** *p* < .001, * *p* < .05
