# Supplementary material for: Why are some countries rich and others poor? development and validation of the attributions for Cross-Country Inequality Scale (ACIS)
Source: PLoS One. 2024 Feb 27;19(2):e0298222. doi: 10.1371/journal.pone.0298222 (PMC10898736; doi:10.1371/journal.pone.0298222)
Supplement: S8 Table — (DOCX) [file pone.0298222.s009.docx]

**Table S8.** Correlations for the South African sample (Study 2; n = 248).

| **Variable** | | **α** | **1** | **2** | **3** | **4** | **5** | **6** | **7** | **8** | **9** | **10** | **11** | **12** | **13** | **14** | **15** | **16** | **17** | **18** | **19** | **20** | **21** | **22** | **23** | **24** |
| --- | --- | --- | --- | --- | --- | --- | --- | --- | --- | --- | --- | --- | --- | --- | --- | --- | --- | --- | --- | --- | --- | --- | --- | --- | --- | --- |
| **1** | **Rich countries** | .91 |  |  |  |  |  |  |  |  |  |  |  |  |  |  |  |  |  |  |  |  |  |  |  |  |
| **2** | **Poor countries** | .79 | -.26** |  |  |  |  |  |  |  |  |  |  |  |  |  |  |  |  |  |  |  |  |  |  |  |
| **3** | **Fate** | .69 | -.01 | .37** |  |  |  |  |  |  |  |  |  |  |  |  |  |  |  |  |  |  |  |  |  |  |
| **4** | **Inequality perception** |  | .43** | -.08 | -.07 |  |  |  |  |  |  |  |  |  |  |  |  |  |  |  |  |  |  |  |  |  |
| **5** | **Redistribution** |  | .40** | -.13* | .12 | .23** |  |  |  |  |  |  |  |  |  |  |  |  |  |  |  |  |  |  |  |  |
| **6** | **Migration** |  | .35** | -.06 | .05 | .19** | .34** |  |  |  |  |  |  |  |  |  |  |  |  |  |  |  |  |  |  |  |
| **7** | **Unfairness** |  | .37** | -.30** | -.08 | .22** | .26** | .18** |  |  |  |  |  |  |  |  |  |  |  |  |  |  |  |  |  |  |
| **8** | **Morality** |  | .48** | -.21** | -.06 | .29** | .34** | .29** | .26** |  |  |  |  |  |  |  |  |  |  |  |  |  |  |  |  |  |
| **9** | **Moral outrage** |  | .57** | -.24** | .03 | .25** | .46** | .29** | .21** | .53** |  |  |  |  |  |  |  |  |  |  |  |  |  |  |  |  |
| **10** | **Country SES** |  | .01 | .14* | .05 | -.05 | .15* | .04 | -.14* | .00 | -.06 |  |  |  |  |  |  |  |  |  |  |  |  |  |  |  |
| **11** | **Need for**  **institutions** |  | .16** | .05 | .11 | .06 | .17** | .37** | -.03 | .12* | .12 | .08 |  |  |  |  |  |  |  |  |  |  |  |  |  |  |
| **12** | **Trust in**  **institutions** |  | -.26** | .14* | .11 | -.15* | .09 | .08 | -.20** | -.19** | -.02 | -.04 | .32** |  |  |  |  |  |  |  |  |  |  |  |  |  |
| **13** | **Horizontal trust** |  | -.21** | -.02 | -.10 | -.05 | -.11 | -.09 | -.07 | -.07 | -.14* | .08 | -.02 | .03 |  |  |  |  |  |  |  |  |  |  |  |  |
| **14** | **Zero sum beliefs** | .86 | .57** | -.13* | .17** | .22** | .30** | .24** | .17** | .25** | .39** | -.02 | .17** | -.14* | -.18** |  |  |  |  |  |  |  |  |  |  |  |
| **15** | **Meritocracy** | .79 | -.15* | .45** | .16* | -.04 | -.04 | .01 | -.24** | -.16** | -.18** | .18** | .08 | .13 | .05 | -.09 |  |  |  |  |  |  |  |  |  |  |
| **16** | **SDO** | .58 | -.41** | .19** | .26** | -.17** | -.16* | -.16* | -.21** | -.39** | -.33** | -.03 | -.09 | .03 | .14* | -.10 | .11 |  |  |  |  |  |  |  |  |  |
| **17** | **ESJ** | .61 | -.37** | .43** | .36** | -.27** | -.14* | -.06 | -.17** | -.33** | -.30** | .06 | -.01 | .23** | .02 | -.12 | .29** | .39** |  |  |  |  |  |  |  |  |
| **18** | **Country mobility** | .49 | -.29** | .06 | -.14* | -.10 | -.23** | -.15* | -.14* | -.21** | -.19** | .05 | -.09 | -.00 | .01 | -.31** | .13* | .10 | -.05 |  |  |  |  |  |  |  |
| **19** | **Self-identification country** |  | .14* | -.12 | -.14* | .06 | .07 | .12 | .07 | .08 | .07 | -.05 | .16* | .14* | .07 | -.00 | .04 | -.20** | -.08 | .12 |  |  |  |  |  |  |
| **20** | **Self-identification world** |  | -.12 | .13* | -.15* | -.02 | -.04 | .02 | -.07 | -.06 | -.10 | -.01 | -.05 | .14* | .14* | -.21** | .13* | .06 | -.02 | .14* | .32** |  |  |  |  |  |
| **21** | **SSES** |  | -.13* | .14* | -.01 | -.15* | -.12 | -.11 | -.16* | -.15* | -.14* | .18** | .03 | -.01 | .13* | -.14* | .03 | .02 | -.00 | .18** | .01 | .22** |  |  |  |  |
| **22** | **Political orientation** |  | -.17** | .10 | .10 | -.13* | -.25** | -.07 | -.13* | -.22** | -.11 | -.00 | -.01 | -.05 | .15* | -.01 | .11 | .24** | .18** | .08 | -.02 | .01 | .01 |  |  |  |
| **23** | **Age** |  | -.06 | -.09 | -.15* | .12 | -.07 | -.19** | -.05 | -.01 | -.04 | .09 | -.07 | -.05 | .16* | -.09 | -.06 | .02 | -.04 | .11 | -.09 | -.04 | -.01 | .00 |  |  |
| **24** | **Gender** |  | .05 | -.07 | -.06 | .04 | .06 | -.03 | .07 | .01 | .07 | -.18** | -.04 | .07 | -.08 | .13* | -.20** | -.12 | -.07 | .02 | -.15* | -.16* | .00 | .02 | .09 |  |
| **25** | **Education** |  | .04 | -.07 | -.17** | .01 | -.04 | .06 | .01 | .05 | -.03 | -.05 | -.10 | -.09 | -.03 | -.10 | -.02 | -.13* | -.07 | -.01 | .21** | .11 | -.01 | .00 | -.01 | -.07 |

*Note.* SDO = Social Dominance Orientation; ESJ = Economic System Justification; SSES = Subjective Socioeconomic Status. ** *p* < .001, * *p* < .05
