# Supplementary material for: Why are some countries rich and others poor? development and validation of the attributions for Cross-Country Inequality Scale (ACIS)
Source: PLoS One. 2024 Feb 27;19(2):e0298222. doi: 10.1371/journal.pone.0298222 (PMC10898736; doi:10.1371/journal.pone.0298222)
Supplement: S9 Table — (DOCX) [file pone.0298222.s010.docx]

**Table S9.** Correlations for the British sample (Study 2; n = 249).

| **Variable** | | **α** | **1** | **2** | **3** | **4** | **5** | **6** | **7** | **8** | **9** | **10** | **11** | **12** | **13** | **14** | **15** | **16** | **17** | **18** | **19** | **20** | **21** | **22** | **23** | **24** |
| --- | --- | --- | --- | --- | --- | --- | --- | --- | --- | --- | --- | --- | --- | --- | --- | --- | --- | --- | --- | --- | --- | --- | --- | --- | --- | --- |
| **1** | **Rich countries** | .93 |  |  |  |  |  |  |  |  |  |  |  |  |  |  |  |  |  |  |  |  |  |  |  |  |
| **2** | **Poor countries** | .84 | -.38** |  |  |  |  |  |  |  |  |  |  |  |  |  |  |  |  |  |  |  |  |  |  |  |
| **3** | **Fate** | .67 | -.14* | .25** |  |  |  |  |  |  |  |  |  |  |  |  |  |  |  |  |  |  |  |  |  |  |
| **4** | **Inequality perception** |  | .48** | -.42** | -.15* |  |  |  |  |  |  |  |  |  |  |  |  |  |  |  |  |  |  |  |  |  |
| **5** | **Redistribution** |  | .39** | -.33** | -.16* | .51** |  |  |  |  |  |  |  |  |  |  |  |  |  |  |  |  |  |  |  |  |
| **6** | **Migration** |  | .42** | -.44** | -.27** | .36** | .44** |  |  |  |  |  |  |  |  |  |  |  |  |  |  |  |  |  |  |  |
| **7** | **Unfairness** |  | -.50** | .40** | .21** | -.61** | -.45** | -.34** |  |  |  |  |  |  |  |  |  |  |  |  |  |  |  |  |  |  |
| **8** | **Morality** |  | .47** | -.35** | -.20** | .50** | .47** | .37** | -.57** |  |  |  |  |  |  |  |  |  |  |  |  |  |  |  |  |  |
| **9** | **Moral outrage** |  | .47** | -.39** | -.15* | .54** | .62** | .40** | -.56** | .56** |  |  |  |  |  |  |  |  |  |  |  |  |  |  |  |  |
| **10** | **Country SES** |  | .16* | -.13* | -.01 | .07 | .01 | .02 | -.12 | .08 | .12 |  |  |  |  |  |  |  |  |  |  |  |  |  |  |  |
| **11** | **Need for**  **institutions** |  | .36** | -.29** | -.09 | .36** | .29** | .34** | -.21** | .22** | .28** | .06 |  |  |  |  |  |  |  |  |  |  |  |  |  |  |
| **12** | **Trust in**  **institutions** |  | .04 | -.24** | -.12 | .14 | .25** | .28** | .06 | -.01 | .02 | .02 | .38** |  |  |  |  |  |  |  |  |  |  |  |  |  |
| **13** | **Horizontal trust** |  | -.03 | -.13* | -.08 | .08 | .13* | .07 | -.02 | -.02 | .00 | -.03 | .10 | .21** |  |  |  |  |  |  |  |  |  |  |  |  |
| **14** | **Zero sum beliefs** | .87 | .63** | -.23** | -.11 | .34** | .34** | .28** | -.35** | .32** | .41** | .04 | .27** | .04 | -.06 |  |  |  |  |  |  |  |  |  |  |  |
| **15** | **Meritocracy** | .87 | -.29** | .48** | .19** | -.27** | -.27** | -.27** | .35** | -.33** | -.35** | -.05 | -.04 | -.01 | -.00 | -.14* |  |  |  |  |  |  |  |  |  |  |
| **16** | **SDO** | .79 | -.44** | .41** | .24** | -.58** | -.41** | -.35** | .43** | -.42** | -.45** | .05 | -.32** | -.20** | -.03 | -.29** | .22** |  |  |  |  |  |  |  |  |  |
| **17** | **ESJ** | .81 | -.54** | .58** | .28** | -.54** | -.41** | -.38** | .57** | -.51** | -.52** | -.08 | -.30** | .00 | -.10 | -.28** | .60** | .56** |  |  |  |  |  |  |  |  |
| **18** | **Country mobility** | .56 | -.33** | .23** | .02 | -.17** | -.24** | -.14* | .18** | -.17** | -.18** | -.02 | -.23** | -.15* | -.00 | -.42** | .11 | .10 | .13* |  |  |  |  |  |  |  |
| **19** | **Self-identification country** |  | -.36** | .32** | .21** | -.19** | -.24** | -.21** | .25** | -.22** | -.33** | -.05 | -.10 | .13 | .04 | -.20** | .36** | .18** | .44** | .15* |  |  |  |  |  |  |
| **20** | **Self-identification world** |  | .02 | -.21** | -.08 | .09 | .22** | .17** | -.18** | .12 | .15* | .13* | .16* | .21** | .24** | -.04 | -.23** | -.11 | -.21** | .08 | .16* |  |  |  |  |  |
| **21** | **SSES** |  | -.08 | -.05 | .15* | -.05 | -.01 | -.05 | .03 | .05 | -.08 | .06 | .03 | .19** | .12 | -.12 | -.01 | .12 | .10 | -.00 | .07 | .29** |  |  |  |  |
| **22** | **Political orientation** |  | -.48** | .45** | .23** | -.42** | -.44** | -.42** | .46** | -.43** | -.48** | .02 | -.26** | .07 | -.07 | -.25** | .49** | .40** | .67** | .17** | .47** | -.12 | .10 |  |  |  |
| **23** | **Age** |  | -.11 | .10 | .12 | -.08 | -.04 | -.17** | .11 | -.12 | -.11 | -.02 | .05 | .09 | .25** | -.15* | .08 | .05 | .11 | .14* | .22** | .12 | .12 | .20** |  |  |
| **24** | **Gender** |  | .08 | -.21** | -.06 | .21** | .15* | .08 | -.10 | .05 | .17** | -.04 | .02 | .05 | -.11 | .15* | -.19** | -.22** | -.14* | .03 | -.06 | -.01 | -.06 | -.16** | -.15* |  |
| **25** | **Education** |  | .09 | -.19** | -.08 | .15* | .19** | .06 | -.19** | .20** | .17** | .04 | .08 | .02 | .06 | .02 | -.21** | -.06 | -.21** | -.05 | -.11 | .21** | .24** | -.19** | -.06 | .14* |

*Note.* SDO = Social Dominance Orientation; ESJ = Economic System Justification; SSES = Subjective Socioeconomic Status. ** *p* < .001, * *p* < .05
