# Supplementary material for: Why are some countries rich and others poor? development and validation of the attributions for Cross-Country Inequality Scale (ACIS)
Source: PLoS One. 2024 Feb 27;19(2):e0298222. doi: 10.1371/journal.pone.0298222 (PMC10898736; doi:10.1371/journal.pone.0298222)
Supplement: S10 Table — (DOCX) [file pone.0298222.s011.docx]

**Table S10.** Regression results of causal attributions on inequality appraisals (Study 2; N = 736).

|  | **Inequality Perception** | | | | | | **Redistribution** | | | | | |
| --- | --- | --- | --- | --- | --- | --- | --- | --- | --- | --- | --- | --- |
|  | **Model 1 (R^2^ adjusted = .226)** | | | **Model 2 (R^2^ adjusted = .221)** | | | **Model 1 (R^2^ adjusted = .145)** | | | **Model 2 (R^2^ adjusted = .141)** | | |
| **Predictors** | **Beta** | **95% CI** | ***p*** | **Beta** | **95% CI** | ***p*** | **Beta** | **95% CI** | ***p*** | **Beta** | **95% CI** | ***p*** |
| (Intercept) | 0.11 | -0.02 – 0.23 | **<.001** | 3.18 | 0.04 – 0.33 | **<.001** | 0.12 | -0.02 – 0.25 | **.002** | 0.19 | 0.05 – 0.34 | **.002** |
| Rich countries | 0.41 | 0.34 – 0.48 | **<.001** | 0.41 | 0.34 – 0.48 | **<.001** | 0.33 | 0.26 – 0.41 | **<.001** | 0.33 | 0.26 – 0.40 | **<.001** |
| Poor countries | -0.10 | -0.18 – -0.02 | **.018** | -0.07 | -0.17 – -0.01 | **.032** | -0.12 | -0.20 – -0.03 | **.007** | -0.10 | -0.19 – -0.02 | **.018** |
| Fate | -0.07 | -0.13 – 0.00 | .057 | -0.05 | -0.13 – 0.00 | .065 | 0.04 | -0.03 – 0.11 | .249 | 0.05 | -0.03 – 0.12 | .213 |
| Country [ita] | -0.14 | -0.31 – 0.04 | .134 | -0.08 | -0.29 – 0.07 | .216 | -0.16 | -0.35 – 0.02 | .089 | -0.14 | -0.33 – 0.04 | .130 |
| Country [uk] | -0.19 | -0.37 – -0.00 | **.046** | -0.15 | -0.41 – -0.01 | **.044** | -0.19 | -0.38 – 0.01 | .061 | -0.17 | -0.38 – 0.04 | .118 |
| Gender [male] |  |  |  | -0.10 | -0.28 – -0.02 | **.027** |  |  |  | -0.17 | -0.31 – -0.04 | **.012** |
| Gender [non-binary] |  |  |  | -0.42 | -1.25 – 0.07 | .080 |  |  |  | -0.24 | -0.94 – 0.46 | .498 |
| Age |  |  |  | 0.00 | -0.05 – 0.10 | .500 |  |  |  | -0.00 | -0.09 – 0.08 | .927 |
|  | *F*(3,727) = 2.59, *p* .052 | | | | | | F(3,727) = 2.19, p .088 | | | | | |
|  | **Migration** | | | | | | **Unfairness** | | | | | |
|  | **Model 1 (R^2^ adjusted = .241)** | | | **Model 2 (R^2^ adjusted = .223)** | | | **Model 1 (R^2^ adjusted = .193)** | | | **Model 2 (R^2^ adjusted = .194)** | | |
|  | **Beta** | **95% CI** | ***p*** | **Beta** | **95% CI** | ***p*** | **Beta** | **95% CI** | ***p*** | **Beta** | **95% CI** | ***p*** |
| (Intercept) | 0.37 | 0.25 – 0.50 | **<.001** | 0.31 | 0.17 – 0.45 | **<.001** | -0.06 | -0.19 – 0.07 | **<.001** | -0.07 | -0.21 – 0.08 | **<.001** |
| Rich countries | 0.32 | 0.25 – 0.39 | **<.001** | 0.30 | 0.23 – 0.37 | **<.001** | 0.35 | 0.28 – 0.43 | **<.001** | 0.35 | 0.28 – 0.42 | **<.001** |
| Poor countries | -0.15 | -0.23 – -0.07 | **<.001** | -0.14 | -0.22 – -0.07 | **<.001** | -0.18 | -0.26 – -0.10 | **<.001** | -0.18 | -0.26 – -0.10 | **<.001** |
| Fate | -0.03 | -0.09 – 0.04 | .452 | -0.03 | -0.10 – 0.04 | .422 | -0.04 | -0.11 – 0.02 | .206 | -0.05 | -0.12 – 0.02 | .200 |
| Country [ita] | -0.50 | -0.67 – -0.32 | **<.001** | -0.52 | -0.69 – -0.34 | **<.001** | 0.09 | -0.09 – 0.27 | .327 | 0.09 | -0.09 – 0.27 | .337 |
| Country [uk] | -0.62 | -0.81 – -0.44 | **<.001** | -0.43 | -0.63 – -0.23 | **<.001** | 0.09 | -0.10 – 0.27 | .370 | 0.15 | -0.06 – 0.36 | .159 |
| Gender [male] |  |  |  | -0.00 | -0.13 – 0.13 | .955 |  |  |  | -0.02 | -0.15 – 0.12 | .806 |
| Gender [non-binary] |  |  |  | 0.15 | -0.50 – 0.81 | .649 |  |  |  | -0.20 | -0.87 – 0.48 | .567 |
| Age |  |  |  | -0.17 | -0.25 – -0.10 | **<.001** |  |  |  | -0.06 | -0.14 – 0.02 | .173 |
|  | F(3,727) = 6.59, p <.001 | | | | | | F(3,727) = 0.73, p = .532 | | | | | |
|  | **Moralization** | | | | | | **Moral Outrage** | | | | | |
|  | **Model 1 (R^2^ adjusted = .234)** | | | **Model 2 (R^2^ adjusted = .235)** | | | **Model 1 (R^2^ adjusted = .291)** | | | **Model 2 (R^2^ adjusted = .284)** | | |
|  | **Beta** | **95% CI** | ***p*** | **Beta** | **95% CI** | ***p*** | **Beta** | **95% CI** | ***p*** | **Beta** | **95% CI** | ***p*** |
| (Intercept) | -0.02 | -0.14 – 0.11 | **<.001** | 0.01 | -0.13 – 0.15 | **<.001** | 0.15 | 0.03 – 0.27 | **<.001** | 0.23 | 0.09 – 0.36 | **<.001** |
| Rich countries | 0.41 | 0.34 – 0.48 | **<.001** | 0.41 | 0.34 – 0.48 | **<.001** | 0.43 | 0.36 – 0.50 | **<.001** | 0.43 | 0.36 – 0.49 | **<.001** |
| Poor countries | -0.12 | -0.20 – -0.04 | **.004** | -0.11 | -0.19 – -0.03 | **.006** | -0.14 | -0.21 – -0.06 | **.001** | -0.12 | -0.20 – -0.04 | **.002** |
| Fate | -0.07 | -0.14 – -0.00 | **.043** | -0.07 | -0.14 – -0.00 | **.045** | -0.02 | -0.09 – 0.05 | .553 | -0.02 | -0.08 – 0.05 | .619 |
| Country [ita] | 0.12 | -0.05 – 0.30 | .167 | 0.13 | -0.04 – 0.31 | .137 | -0.07 | -0.24 – 0.11 | .451 | -0.05 | -0.22 – 0.12 | .555 |
| Country [uk] | -0.07 | -0.25 – 0.11 | .444 | -0.06 | -0.27 – 0.14 | .532 | -0.39 | -0.57 – -0.21 | **<.001** | -0.34 | -0.53 – -0.14 | **.001** |
|  |  |  |  |  |  |  |  |  |  |  |  |  |
| Gender [male] |  |  |  | -0.06 | -0.19 – 0.07 | .371 |  |  |  | -0.19 | -0.31 – -0.06 | **.003** |
| Gender [non-binary] |  |  |  | -0.38 | -1.04 – 0.28 | .262 |  |  |  | -0.19 | -0.83 – 0.44 | .547 |
| Age |  |  |  | -0.00 | -0.08 – 0.08 | .946 |  |  |  | -0.04 | -0.11 – 0.04 | .308 |
|  | *F*(3,727) = 0.635, *p* = .592 | | | | | | *F*(3,727) = 3.42, *p* = .017 | | | | | |

*Notes.* Country reference level is South Africa. Gender reference level is female
